# The ins and outs of metal homeostasis by the root nodule actinobacterium *Frankia*

**DOI:** 10.1186/1471-2164-15-1092

**Published:** 2014-12-12

**Authors:** Teal R Furnholm, Louis S Tisa

**Affiliations:** Department of Molecular, Cellular and Biomedical Sciences, University of New Hampshire, Durham, NH USA

**Keywords:** Actinobacteria, Actinorhizal symbiosis, Bioremediation, Comparative genomics, Metal homeostasis, Metal tolerance

## Abstract

**Background:**

*Frankia* are actinobacteria that form a symbiotic nitrogen-fixing association with actinorhizal plants, and play a significant role in actinorhizal plant colonization of metal contaminated areas. Many *Frankia* strains are known to be resistant to several toxic metals and metalloids including Pb^2+^, Al^+3^, SeO_2_, Cu^2+^, AsO_4_, and Zn^2+^. With the availability of eight *Frankia* genome databases, comparative genomics approaches employing phylogeny, amino acid composition analysis, and synteny were used to identify metal homeostasis mechanisms in eight *Frankia* strains. Characterized genes from the literature and a meta-analysis of 18 heavy metal gene microarray studies were used for comparison.

**Results:**

Unlike most bacteria, *Frankia* utilize all of the essential trace elements (Ni, Co, Cu, Se, Mo, B, Zn, Fe, and Mn) and have a comparatively high percentage of metalloproteins, particularly in the more metal resistant strains. Cation diffusion facilitators, being one of the few known metal resistance mechanisms found in the *Frankia* genomes, were strong candidates for general divalent metal resistance in all of the *Frankia* strains. Gene duplication and amino acid substitutions that enhanced the metal affinity of CopA and CopCD proteins may be responsible for the copper resistance found in some *Frankia* strains. CopA and a new potential metal transporter, DUF347, may be involved in the particularly high lead tolerance in *Frankia*. Selenite resistance involved an alternate sulfur importer (CysPUWA) that prevents sulfur starvation, and reductases to produce elemental selenium. The pattern of arsenate, but not arsenite, resistance was achieved by *Frankia* using the novel arsenite exporter (AqpS) previously identified in the nitrogen-fixing plant symbiont *Sinorhizobium meliloti*. Based on the presence of multiple tellurite resistance factors, a new metal resistance (tellurite) was identified and confirmed in *Frankia*.

**Conclusions:**

Each strain had a unique combination of metal import, binding, modification, and export genes that explain differences in patterns of metal resistance between strains. *Frankia* has achieved similar levels of metal and metalloid resistance as bacteria from highly metal-contaminated sites. From a bioremediation standpoint, it is important to understand mechanisms that allow the endosymbiont to survive and infect actinorhizal plants in metal contaminated soils.

**Electronic supplementary material:**

The online version of this article (doi:10.1186/1471-2164-15-1092) contains supplementary material, which is available to authorized users.

## Background

*Frankia* is a soil dwelling diazotrophic actinobacteria that forms a symbiosis with a variety of woody dicots, primarily of the nitrogen-fixing clade of Eurosids [1, 2]. Actinorhizal plants are found worldwide in a broad range of ecological conditions [3]. The symbiosis with *Frankia* allows these actinorhizal host plants to colonize harsh environmental terrains including highly contaminated or nutrient-poor soils [4]. *Frankia* cultures exhibit elevated levels of tolerance to various heavy metals including Pb^2+^, Al^3+^, SeO_2_^3−^, Cu^2+^, AsO_4_, and Zn^2+^[5]. The levels of tolerance to several heavy metals by some *Frankia* strains are even greater than those of *Cupriavidus metallidurans*, a well-characterized metal-resistant β-proteobacterium isolated from metal-contaminated sediment [6]. As a potential consequence of their association with plants that frequently grow in poor soils with low buffering capacity, *Frankia* may be exposed to high levels of metals. Under these low-buffering-soil conditions, metals may be freely solubilized from the soil substrate through the action of organic acids, phenolics, and protons produced from both plant and microbial communities [7].

Nearly a quarter of all proteins require a metal cofactor, many of which are toxic at elevated levels [8]. *Frankia* are highly versatile as a saprophyte, plant symbiont, diazotroph, and producer of secondary metabolites, and therefore require many essential metals for growth [9]. The effects of metals on *Frankia* physiology have been investigated in culture [10, 11], *in planta*[12, 13], and in the field [14–16]. However, little is known about the molecular details and mechanisms of metal resistance and homeostasis by these actinobacteria.

Generally, bacteria have a combination of low and high affinity permeases and ATP-driven metal importers for each of the essential metals [17]. Toxic metals (mercury, lead, cadmium, arsenic, etc.) are often unintentionally imported with the beneficial ones due to similar atomic and aqueous divalent ion radii [18, 19]. This ion mimicry adds a layer of complexity as cells must not deplete reserves of beneficial metals while exporting their toxic counterparts. Metal export occurs primarily through four mechanisms: (1) cation diffusion facilitators (CDF), (2) resistance-nodulation-cell division (RND) complexes, (3) P-type ATPases, and (4) metal-specific efflux permeases [20]. Many multi-gene transporters are found in a single transcriptional unit (operon), or a separately transcribed functional unit (cassette). An intricate system of metal import, binding, storage, metal cofactor handling, efflux, and cellular redox maintenance mechanisms is needed to maintain metal homeostasis [18, 21]. This complex system must be responsive to external growth conditions, metabolic state, and various cellular activities (e.g. nitrogen fixation), making metal homeostasis one of the most complex biological processes.

Recent sequencing of several *Frankia* genomes has provided new insight on the physiology and phylogeny of *Frankia*[22]. The most striking difference among the first three sequenced *Frankia* genomes was their sizes, which varied from 5.43 Mbp for a narrow host range *Casuarina* strain (*Frankia sp.* strain CcI3) to 9.04 Mbp for a broad host range *Elaeagnus* strain (EAN1pec) [23]. In recent years, several more *Frankia* strains have been sequenced [24–31]. Analysis of these genomes confirmed that the *Frankia* genome size correlates positively to host specificity and biogeography ranges [22, 23]. Presently, genome sequences are available for all four *Frankia* lineages: Cluster 1 “medium and narrow host range” strains CcI3, ACN14a, CcI6, BMG5.23, Thr, and QA3 [23, 26, 29–31]; Cluster 2 “uncultured” *Frankia datiscae* Dg1 [25]; Cluster 3 “broad host range” strains EAN1pec, EUN1f, BMG5.12 and BCU110501 [23, 24, 27]; and Cluster 4 “atypical” strains EuI1c, CN3 and DC12 [22, 24]. Atypical *Frankia* strains used in this study are unable to fix nitrogen, and two (strains CN3 and DC12) are unable to re-infect their host plant. These databases are providing a wealth of information on secondary metabolism, stress tolerance, symbiosis and nitrogen fixation, having been used successfully in genome mining [32, 33], comparative genomics [23, 34–37], transcriptomics [38–40] and proteomics approaches [41–44].

For this study, the genomes from the cultured *Frankia* strains CcI3, ACN14a, QA3, EAN1pec, EuI1c, EUN1f, DC12 and CN3 were used as the databases. These strains represent three of the four *Frankia* lineages, have large genome size ranges (5.43 - 9.97 Mbp), and have diverse levels of association with host plants. In this study, we used bioinformatics and comparative genomics approaches to identify metal homeostasis and toxic metal resistance mechanisms in *Frankia*.

## Results and discussion

### Part 1: Beneficial metals and metal homeostasis

**Figure 1:**
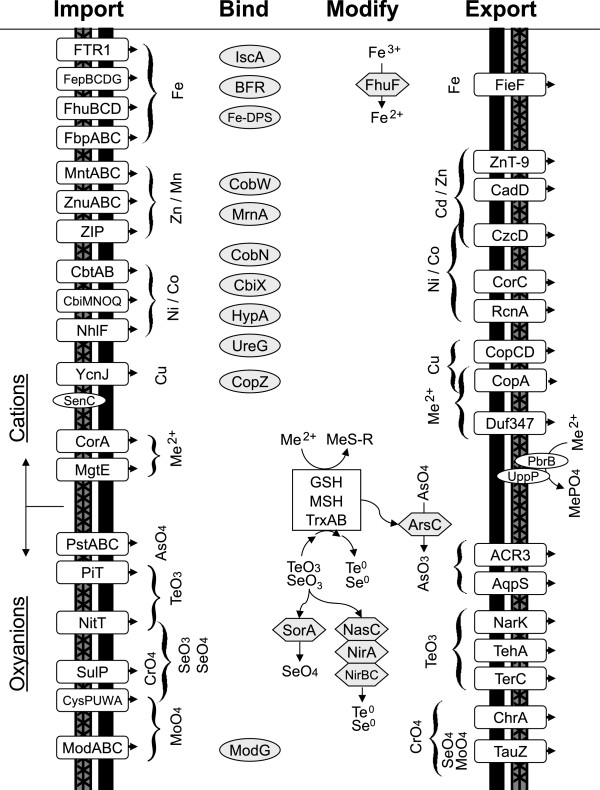
**Overview of metal homeostasis mechanisms in** ***Frankia.*** Schematic diagram of known and putative metal homeostasis systems in *Frankia*. Symbols for proteins involved in metal ion uptake transporters, chaperones, modification enzymes, efflux transporters, and surface binding protein and efflux systems are shown (left to right) with arrows to indicate the flow of metals through the cell. Evidence for displayed mechanisms can be found in Additional file 10. Gene counts for displayed mechanisms are shown in Table 2.

In the absence of genetic tools to assess gene function in *Frankia*, comparative genomics provides a means of exploring *Frankia* metal homeostasis and resistance capabilities. A combination of bioinformatics techniques were used to identify metal homeostasis mechanisms in *Frankia* (Figure 1) including: meta-analysis of 16 gene array studies to identify conserved mechanisms (Additional file 1), phylogenetic grouping with characterized metal homeostasis mechanisms (Additional files 2, 3, 4, 5, 6, 7) gene neighborhood synteny comparison (Additional file 8), and the presence of conserved metal binding residues in protein alignments (Additional file 9). From these analyses, several groups of potential metal homeostasis mechanisms were identified (Additional file 10). Relevance of metal resistance mechanisms was correlated to previously established metal and metalloid tolerance levels [5]. Figure 1 shows a composite diagram of the *Frankia* resistance/tolerance mechanisms, while the profiles for the individual *Frankia* genomes are found in the supplemental materials (Additional files 11, 12, 13, 14, 15, 16, 17, 18). Discussed below are the unique combinations of import, chaperone, modification, and export mechanisms involved in metal homeostasis and toxic metal resistance in eight sequenced *Frankia* strains.

### Iron homeostasis

Iron, zinc, and manganese are essential to most, if not all, organisms [45–47]. Analysis of the *Frankia* genomes revealed hundreds of genes that encode proteins requiring iron (Table 1). Interestingly, the more metal-resistant *Frankia* strains and *C. metallidurans* had significantly higher proportion of iron metalloproteins than other bacteria (Table 1). Three types of iron importers were identified in the *Frankia* genomes: (1) a high-affinity permease, (2) iron-siderophore ABC cassettes, and (3) Fe^3+^ ABC cassettes. A closely related (33% identity) homolog of the ferrous iron transporter (FTR1) from *Saccharomyces cerevisiae* was found present in all of the *Frankia* genomes (Table 2). The *Frankia* FTR1 homologs were in a possible operon with an iron-transport lipoprotein and a Dyp-type haem peroxidase. These proteins may function collectively for iron assimilation.

**Table 1:** **Predicted number of metalloproteins in** ***Frankia*** **and other bacteria**

Metal	[Dom #]	***CcI3***	***ACN***	***QA3***	***EAN1***	***EUN***	***EuI1***	***CN3***	***DC12***	B sub	***C met***	***E col***	***H pyl***	***M tub***
Co	[6]	7	13	12	9	13	15	15	11	11	**26**	13	6	8
Cu	[31]	27	28	34	36	41	31	42	29	23	**72**	17	6	16
Fe	[174]	245	279	328	400	**457**	**445**	**514**	275	179	**398**	227	77	188
Mo	[15]	7	18	15	18	20	16	20	11	8	**28**	**24**	2	12
Mn	[54]	74	102	117	**156**	144	**167**	**185**	91	72	102	76	25	**99**
Ni	[17]	25	35	**39**	38	32	30	28	24	13	29	20	**14**	14
Zn	[230]	174	279	336	**388**	401	**422**	**483**	264	156	305	168	58	215
Genome a		4621	6723	6546	7250	7833	7262	8412	5933	4354	6430	4427	1749	4062
% MPs		12.1	11.2	13.5	14.4	14.1	**15.5**	**15.3**	11.9	10.6	**14.9**	12.3	10.7	13.6
% MR		1.06	1.06	1.06	1.06	1.06	1.06	1.06	1.06	1.06	1.06	1.06	1.06	1.06

Proteins with metal ion binding properties were identified using Gene Ontology function search through the Joint Genome Institute – Integrated Microbial Genomes website (https://img.jgi.doe.gov). Identified protein domains for the metalloproteins were used to find orthologs in *Frankia* and other bacteria including: *Bacillus subtilis subtilis 168 (B sub), Cupriavidus metallidurans CH34 (C met), Escherichia coli K12- W3110 (E col), Helicobacter pylori B8 (H pyl), Mycobacterium tuberculosis H37Rv (M tub),* and *Streptomyces viridochromogenes DSM 40736 (S vir)*. Genomes with significant (>1 S.D. from average) metalloprotein content, normalized to the genome size, are in bold. % MPs = percentage of total metalloproteins in the genome. %MR = percent metal resistance genes (binding, detoxification, and export) in the genome. [Dom #] = Number of unique protein families (pfam) or ortholog clusters (COG) that bind the indicated metal.

a. Total number of protein coding genes in each genome.

**Table 2:** **Abundance of homeostasis mechanisms in** ***Frankia***

Mechanism Counts in Selected Genomes													
	Symbol	***Frankia sp.*** strain CcI3	***Frankia*** alni ACN14a	***Frankia sp.*** strain QA3	***Frankia sp.*** strain EUN1f	***Frankia sp.*** strain EAN1pec	***Frankia sp.*** strain EuI1c	***Frankia sp*** *.* strain CN3	***Frankia sp.*** strain DC12	***S.*** ***viridochromogenes***	***B.*** ***subtilis*** 168	***C.*** ***metallidurans***	***E*** *.* ***coli*** K12- W3110
Import permeases	CbtA	1	1	1	1	1	1	1	1	1	0	0	0
	CorA	1	2	1	1	1	1	2	1	3	1	2	1
	Ftr1	1	1	1	1	1	2	1	1	1	1	1	0
	MgtE	1	1	1	1	1	1	1	1	1	1	0	0
	NhlF	1	1	1	0	0	1	1	1	0	0	1	0
	Pit	0	1	1	1	0	0	1	0	3	1	1	0
	SulP	3	2	2	4	3	2	3	2	3	2	6	1
	YcnJ	1	1	1	1	1	1	1	1	2	1	0	0
	Zip	2	1	0	0	0	0	0	1	0	0	1	1
Import cassettes	CbiMNOQ	0	0	0	1	1	0	0	0	2	1	0	0
	CysPUWA	0	0	0	1	0	1	1	1	0	0	1	1
	FbpABC	1	0	1	2	2	1	0	0	0	0	0	1
	FepBCDG	0	1	1	1	1	1	2	1	2	0	1	1
	FhuBCD	1	2	1	0	1	0	0	1	1	5	1	2
	Mnt/ZnuABC	1	1	1	1	1	1	1	1	1	2	0	1
	Mod ABC	1	1	1	1	1	1	1	1	1	1	1	1
	PstABCS	1	1	1	1	1	1	1	1	1	1	1	1
Chaperones storage	BFR	1	3	3	2	2	2	2	1	1	0	2	1
	CbiX	1	1	1	1	2	1	1	1	3	1	1	0
	ChlD	1	1	3	4	2	2	4	2	0	0	1	0
	ChlI	2	2	2	3	3	2	2	2	1	0	2	1
	CobN	1	1	3	2	1	1	2	3	2	0	0	0
	CobW	0	1	1	1	1	1	1	1	1	1	3	2
	CopZ	1	1	1	3	3	1	2	1	2	1	7	1
	Fe-DPS	1	1	1	1	1	1	1	1	2	2	1	1
	FhuF	3	3	3	3	4	1	1	1	4	0	3	1
	HypA	2	2	2	2	2	1	1	1	1	0	2	2
	HypB	2	2	2	1	1	0	1	0	1	0	2	1
	IscA	2	2	2	2	2	1	1	1	1	1	2	4
	ModG	1	2	2	2	2	0	0	0	1	0	1	1
	MrnA	0	1	1	0	1	1	1	0	1	0	0	0
	SenC	2	3	3	3	2	1	1	2	1	1	3	0
	UreG	1	1	1	1	1	1	1	1	1	0	1	0
Modification	ArsC	0	1	1	0	1	0	0	1	1	3	4	3
	GSH	0	0	0	0	1	1	1	0	0	0	1	1
	MSH	1	1	1	1	1	1	1	1	1	0	0	0
	NasC	1	0	0	0	0	0	2	0	1	1	1	0
	NirB	2	1	1	1	1	1	2	1	2	2	2	1
	NirD	1	1	1	1	1	1	2	1	1	1	1	1
	PbrB	1	1	1	1	1	1	1	1	1	1	3	1
	NirA	1	1	1	1	1	1	1	1	1	0	0	0
	SorA	0	0	0	2	0	1	1	0	0	0	1	0
	TrxA	1	1	2	2	1	2	2	1	2	1	2	1
	TrxB	2	4	5	3	2	5	4	3	3	4	5	1
	UppP	2	3	3	3	3	2	2	1	1	1	1	1
Exporters	ACR3	0	1	1	0	1	0	0	0	1	1	1	0
	AqpS	1	2	2	2	1	3	2	2	3	1	2	2
	CadD	0	0	1	1	1	0	0	0	0	0	0	0
	ChrA	1	0	0	0	0	1	1	2	1	2	5	0
	CopA	1	1	1	2	3	1	3	2	2	1	4	1
	CopC	2	2	2	3	2	2	2	3	2	1	2	1
	CopD	2	2	2	2	2	3	2	2	3	1	2	1
	CorC	2	3	3	3	3	2	2	2	4	5	5	6
	CzcD	1	1	1	1	1	1	1	1	1	1	1	1
	DUF347	2	4	1	2	1	3	3	3	0	0	7	0
	FieF	1	1	0	0	1	1	1	1	1	3	1	1
	NarK	1	1	1	1	1	2	2	1	1	2	3	2
	RcnA	0	0	0	1	1	0	1	0	0	0	0	1
	TauZ	0	0	0	0	0	1	1	1	0	0	2	1
	TehA	0	0	0	0	0	1	0	0	1	0	0	1
	TerC	1	1	1	1	1	1	1	1	1	3	4	4
	ZnT-9	1	2	2	1	1	1	2	1	2	0	0	0

Counts indicate the total number of instances of the mechanism based on protein identifiers listed in Additional file 10. Symbols were assigned either by the closest ortholog in well characterized organisms or most commonly used symbol from the literature.

All of the *Frankia* strains also have at least one ABC cassette, either FhuBCD or FepBCDG that functions to transports iron-siderophore complexes (Table 2). Except for strain CcI3, the *fepBCDG* operons in the *Frankia* genomes contained an enterobactin-type permease (COG4997) and a hydroxamate-type permease (COG0609) (Additional file 2). This heterodimeric iron permease complex may allow the transporter to accept a wider variety of iron chelating substances, such as xenosiderophores of the rhizosphere community, providing a competitive advantage [45, 48]. The presence of an additional iron importing cassette, either citrate or haem-type, was found in several *Frankia* strains (Additional file 10). *Frankia* produces both catecholate and hydroxamate siderophores, making the presence of multiple Fe-siderophore importing ABC cassettes physiologically relevant [49, 50].

During symbiosis, an increased iron acquisition is required by *Frankia* for both nitrogenase activity and hemoglobin production [51]. Only the symbiotic *Frankia* strains contain a third type of iron- ABC cassette (FbpABC), which is used for ferric iron import (Additional file 10). The supplementary high affinity iron transporter may help *Frankia* procure additional iron needed for nitrogenase or for metalloproteins to deal with host-derived phenolic compounds produced during symbiosis [52]. There is a further connection between iron and nitrogen fixation. Nitrogen fixation by *Frankia* and cyanobacteria requires boron [53], and boron facilitates ferric iron transport via FbpABC [54]. Increased oxidative stress occurs from the import of the more toxic ferric-iron and from the host immunity during symbiosis [52, 55]. The nitrogenase enzyme needs iron. but is permanently inactivated once oxidized During nitrogen fixation, the iron co-factored superoxide dismutase (SodF) is up-regulated in *Frankia*[56]. This link between iron and nitrogen-fixation explains why SodF is only found in the N_2_-fixing *Frankia* strains (Additional file 10). The iron chaperone, IscA, is involved in the production of iron-sulfur clusters and was found in all of the *Frankia* genomes (Table 2). The nitrogen-fixing strains have a second copy of the IscA, which is likely used for the Fe-S containing nitrogenase complex [57]. Once inside the cell, iron is removed from siderophores and reduced to ferrous iron by the reductase FhuF [45]. Orthologs of FhuF are 3X more abundant in the nitrogen fixing strains (Table 2), and possibly work in concert with the ferric iron ABC transporters found only in these strains. The ferrous iron is incorporated into proteins by ferrochelatase or placed into bacterioferritin (Bfr) or Dps storage complexes [48]. The ferrous iron efflux protein (FieF) is in the family of cation diffusion facilitators (CDF) which may export both divalent zinc and iron [58]. FieF may help alleviate iron stress in *Frankia* by exporting surplus iron.

### Zinc and manganese homeostasis

All of the *Frankia* genomes have hundreds of zinc and manganese metalloproteins, nearly as many as for iron (Table 1). The necessity of zinc is well established, both as a structural component of macromolecules and as a cofactor in enzymatic reactions, with up to 10% of proteins in a genome requiring a zinc cofactor [59, 60]. Manganese, as an antioxidant in the cytoplasm, helps detoxify oxygen radicals as a cofactor for SodN, and replaces the more toxic iron when cells are under oxidative stress [46, 61]. Zinc and manganese are structural analogs and share transport mechanisms [46]. While the natural resistance-associated macrophage protein (NRAMP)-type manganese permease (MntH) is found in closely related actinomycetes including *Geodermatophilus* and *Mycobacterium*, the *mntH* genes were conspicuously absent in *Frankia* genomes. However, the *Frankia* genomes contained low affinity magnesium permeases, CorA and MgtE (Table 2), which transport several types of divalent cations including zinc [62]. Additionally, *Frankia sp.* strain CcI3 and *F. alni* strain ACN14a contained the zinc-iron permease (ZIP).

All of the *Frankia* genomes possessed high-affinity Mnt/Znu-type ABC transporter. Under low nutrient conditions, this high affinity Mnt/Znu - type ABC cassette functions for both Mn^2+^ and Zn^2+^ uptake, as seen with *M. tuberculosis*[63]. There is little understanding of the appropriate placement of zinc into metalloproteins once it enters the cytoplasm. Certain GTPases are metallochaperones, some of which deliver nickel or cobalt (UreJ/HypB and CobW, respectively), while other proteins (YciC) are suspected to deliver zinc to metalloproteins [64]. The *Frankia* homologs of these GTPases (COG0523) fall phylogenetically between CobW/YjiA and YciC, both of which are zinc chaperones (Additional file 3).

**Figure 2:**
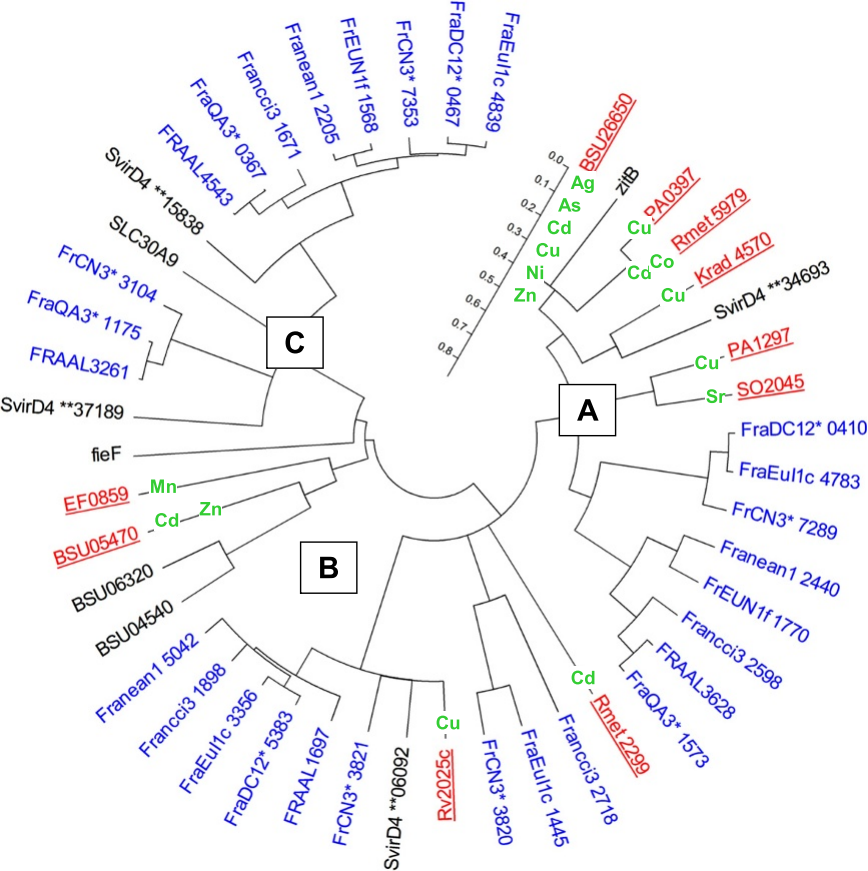
**Phylogeny of cation diffusion facilitators in** ***Frankia.*** Neighbor-joining tree of *Frankia* proteins containing the cation diffusion facilitator (CDF) domain (pfam01545). CDFs used for comparison include the characterized ZnT-9 from *Homo sapiens* (SLC30A9), iron (FieF) and zinc (ZitB) CDFs from *E. coli* K12-W3110, and CDFs up-regulated in the compiled array data (underlined) (see Additional file 1). CDFs of *S. viridochromogenes* and *B. subtilis subtilis* 168, which were used for specific metal resistance comparison in Richards et. al. 2002, were also included. Metals that are up-regulated-specific proteins are displayed on their branches. In locus tags: * = DRAFT, ** = 0101000. The following three types of CDF and identifying regular expression amino acid motifs are shown (“.” indicates any single amino acid, and “.*” indicates unspecified number of amino acids): **A**. CzcD: [QE]........S.....D..H…[DH].*[ST][FWY].{2,3}[RK].*H…D.*[DE].H, **B**. FieF: [FY]G[HY].[RK].E.*D…[SN].....[GA].*R..G.*..P, **C**. ZnT-9: N......K.*HS..D..N.*ED.{24,24}[FWD][ED].

Some *Frankia* strains are resistant up to 8 mM zinc (data not shown), which is a level comparable to the metal resistant *C. metallidurans* isolated from a zinc contaminated site [6]. The absence of zinc-type ATPases (EC:3.6.3.3 and 3.6.3.5) and heavy metal RND (HME-RND) exporters in the *Frankia* genomes indicates their reliance on other resistance mechanisms. In addition to FieF, two more cation diffusion facilitators (CzcD and ZnT-9) were present in all of the *Frankia* genomes and probably contribute to zinc resistance (Figure 2). CzcD is found up-regulated in both gram-positive and gram-negative bacteria for a variety of metal cations (e.g. Zn^2+^, Cd^2+^, Cu^2+^) and, being under control of ArsR, it is also up-regulated by arsenic oxyanions (Additional file 1). The CzcD orthologs found in the *Frankia* genomes are highly similar to those found in the array studies (Figure 2). As one of the few orthologs to known metal export mechanisms, CzcD may contribute significantly to *Frankia* metal resistance. Unlike CzcD, the exact function of the other CDF transporter (ZnT-9) remains elusive [65].

Due to its ionic mimicry of zinc, cadmium likely follows the same route into and out of the cell, being imported through general ion permeases (e.g. CorA) and exported via cation diffusion facilitators [59]. CadD, which is involved in low-level cadmium resistance [66], was present in three *Frankia* genomes, but its contribution to metal resistance is not clear based on metal resistance patterns alone (Table 2). Manganese has low toxicity and there are few known bacterial manganese-specific exporters [46]. Because of its similarity to zinc and iron, *Frankia* probably exports manganese via cation diffusion facilitators, as was demonstrated for *Arabidopsis* and *Streptococcus pneumoniae*[67, 68].

### Nickel and cobalt homeostasis

Several other trace elements act as cofactors for hundreds of metalloproteins, including: nickel [69], cobalt [70], copper [71], molybdenum [72], and selenium [73, 74]. Although these trace elements are used by most organisms, only 17% of them use all five as cofactors [47]. All of the *Frankia* strains appear to utilize all five trace elements (Table 1), with each strain having at least three different metalloenzymes per element (data not shown). Nickel import occurs through active transport via the nickel ABC cassette (NikABCDER) or by high (HoxN/NhlF) and low affinity (CorA and MgtE) permeases [61]. All of the *Frankia* genomes, except strain EUN1f, have a high affinity nickel permease (NhlF) (Table 2). Additionally, the *Frankia* genomes possessed a wide range (4 to 23) of the binding component (*nikA*) genes and multiple combinations of the remaining subunits (*nikBCDE*) (Additional file 10).

Nickel and oligopeptide ABC transporter subunits are highly homologous and multiple nickel/oligopeptide cassettes are frequently present in a genome [69]. Protein sequence alignments showed that oligopeptide (DppA) and nickel transporters of *Escherichia coli* and *Bacillus subtilis* are more closely related to each other than any of the *Frankia* homologs (data not shown), making the separation of these functions in *Frankia* impossible without experimentation. NikA/ DppA homologs are also heme binding, creating a relationship between iron and nickel transport, possibly due to NiFe cofactored enzymes (e.g. hydrogenase) [75]. The nickel-iron link may explain both the large number of NikA homologs, which were directly proportional to the number of bacterioferritins in their respective *Frankia* strains (Additional file 10), and the large number of iron and nickel co-factored proteins in *Frankia* (Tables 1).

Once inside the cell, chaperones, such as UreG and HypAB, bind and insert nickel into the appropriate protein (e.g. urease) [76]. Both of these chaperones were found in all of the *Frankia* genomes (Additional file 3). At elevated levels, nickel becomes toxic due to displacement of cognate metal cofactors in enzymes that facilitate electron transfers [77]. With *B. subtilis*, the cation diffusion facilitator (CzcD) was more highly expressed with nickel exposure over that of any other cation (Additional file 1). *Frankia* strains, which are equally resistant to nickel as *E. coli* and *B. subtilis*[5], have orthologs to CzcD_*Bsub*_ (Figure 2) and so may also use CzcD to export surplus nickel. *Frankia sp.* strain EAN1pec, EUN1f, and CN3 genomes contained a nickel permease that phylogenetically clusters with the nickel and cobalt efflux permease (RcnA) from *E. coli* rather than NhlF (Additional file 4)*.* This transporter provides only a low-level of nickel resistance [78], but may have another function since all of the *Frankia* strains are similarly resistant to Ni^2+^.

Cobalt plays an important role as a cofactor for corrinoids, such as cobalamin (vitamin B12), and several other metalloenzymes including methionine synthase and bromoperoxidase [70]. The cobalt import cassette (CbiMNOQ) is an energy coupled factor (ECF)-type cobalt transporter involved in cobalamin formation [79]. Only *Frankia* EUN1f and EAN1pec genomes possessed CbiMNOQ. The other *Frankia* strains may be obtaining sufficient cobalt via the CbtA, CorA, or MgtE permeases (Table 2). These permeases allow passage of Co^2+^ and other divalent cations [61].

Cobalt toxicity is due to generation of reactive oxygen species and mis-incorporation into iron metalloproteins [70]. To guide correct insertion of cobalt into proteins, all of the *Frankia* genomes possessed both aerobic (CobN) and anaerobic (CbiX) cobalt chaperones. While *Frankia* is not an anaerobe, it can lower cytoplasmic oxygen to protect the nitrogenase complex inside nitrogen-fixing vesicles surrounded by multiple layers of hopanoid lipids [80]. At least two copies of the cobalt exporter (*corC*) gene were found in all of the *Frankia* genomes (Additional file 10). In several different bacteria, this transporter was up-regulated with a variety of di- and tri- valent metals (Additional file 1), and may function as the exporter counterpart to the general metal importer CorA.

### Copper homeostasis

Bacterial cuproenzymes are typically involved in cellular redox cycling reactions, such as oxidation of carbon compounds (e.g. laccase, tyrosinase), electron transport (e.g. cyanins, cytochrome c), and reduction of inorganic oxides (e.g. nitrite reductase, superoxide dismutase) [81]. In Gram-negative bacteria, the use of copper is limited to either the periplasm or cytoplasmic membrane, and so there are few known copper-specific importers [71]. In Gram-positive bacteria, the absence of a periplasm for handling copper necessitated cytoplasmic involvement. This likely drove the evolution of a copper influx permease (YcnJ) and the more copper-resistant cellular thiols (e.g. mycothiol) [82, 83]. The proposed process of copper homeostasis in *Frankia* is as follows: copper import via YcnJ [84], detoxification by metallothioneins (MrnA) [85], chaperoning of copper to cuproenzymes or exporters by CopZ [86], and exported by transporters such as CopA and CopCD [20, 81]. Furthermore, three of *Frankia* strains (DC12, EuI1c, and CN3) are highly resistant to copper at MIC values of 2, 5, and 20 mM, respectively [5]. Studies on copper resistance are complicated due to the presence of multiple resistance mechanisms (CopZA, CopABCD operon, and the CusCBA) [85]. With *Frankia*, mechanisms for copper resistance were not obvious. *Frankia* genomes did not possess a CusCBA system, and all of them have at least one Cu^2+^-ATPase (*copA*) and two *copCD* genes (Table 2). Amino acid substitutions in copies of CopA and CopD are likely contributors to the high levels of copper resistance in the resistant *Frankia* strains (discussed below).

**Figure 3:**
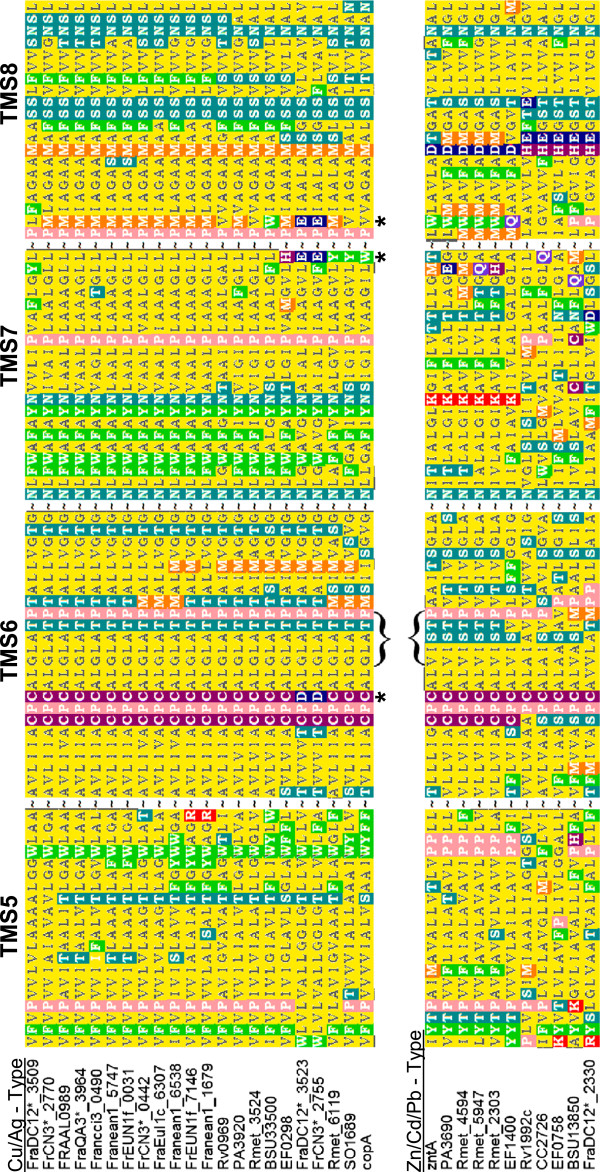
**Protein alignment of transmembrane segments 5 thru 8 of Cu/Ag or Zn/Cd/Pb – type P**_**1B**_ **-ATPases in** ***Frankia.*** Transmembrane segments of *Frankia* heavy metal exporting ATPases (EC: 3.6.3.4 and 3.6.3.5) were aligned to identify amino acid changes that may alter metal specificity. Characterized ATPases from *Escherichia coli* K12-W3110 and those gene products that were up-regulated in the multiple array studies are included for comparison. Brackets indicate conserved amino acids that distinguish Cu/Ag - type from Zn/Cd/Pb - type ATPases. Asterisks indicate aspartate (D) and glutamate (E) amino acid residues in *Frankia* strains CN3 and DC12 that are potentially important for enhanced copper export. Amino acids are colored by characteristic: dark blue are negatively charged (D/E), purple are uncharged metal-binding (C/H), red are positively charged (K/R), pink are cyclic (P), orange are metal-binding and hydrophobic (M), yellow are hydrophobic (L/I/V/M/G/A), green are aromatic (F/W/Y), light blue are polar (S/T/N/Q).

Bacterial P-type ATPases fall into two types: one exports copper and silver (EC:3.6.3.4), the other generally exports lead, zinc, and cadmium (EC:3.6.3.5) [20]. Protein sequence comparison with experimentally verified P-type ATPases indicated that 14 out of 15 *Frankia* ATPases were of the Cu/Ag-type (CopA) (Figure 3). CopA proteins are distinguished by the amino acid motif, CPxALGLATP, found in trans-membrane segment 6, compared to the xPCALVxxTx motif of Pb/Cd/Zn-type ATPases (Figure 3). Several *Frankia* genomes contained duplications of *copA*, but all *copA* genes in copper sensitive strains were on a distinct phylogenetic branch (Additional file 5). Many CopA proteins function as a low rate copper efflux system for cytochrome c oxidase, rather than as a resistance mechanism [87]. Both heme and cytochrome c biosynthesis operons are located in the *copA* gene neighborhood of *Frankia* genomes (Additional file 8). It is possible that most *Frankia* CopA proteins function to provide copper to electron transport chain components, or to maintain low cytoplasmic copper levels during heme synthesis [84].

Two duplicate CopA proteins (FrCN3draft_2755 and FraDC12draft_3523) contained several amino acid substitutions with negatively charged residues (Asp and Glu) and had a His rich N-terminus (Figure 3). These proteins also grouped phylogenetically with well-characterized copper-resistance CopA transporters rather than other *Frankia* Cu^2+^-ATPases (Additional file 5). This evidence would suggest that these Cu^2+^-ATPases may function as copper resistance mechanisms for CN3 and DC12.

YcnJ, was found in each *Frankia* genome (Table 2), and consists of a fused CopC/CopD polypeptide that may function as a copper importer under copper limiting conditions [84]. Another copper transport system is the *copABCD* operon (*copA* here being a multi-copper oxidase not a Cu^2+^-ATPase), which has been implicated in both copper import and export [81, 88, 89]. CopC and CopD are considered a functional unit, with CopC binding copper, possibly for delivery to the copper permease CopD [81]. All *Frankia* strains have both copper importing YcnJ and copper importing/exporting CopCD systems (Additional file 6). In *Frankia*, CopD is a polypeptide with an attached cytochrome C oxidase (Caa3) domain. CopC and SenC may work in tandem with this CopD-Caa3 protein to delivery copper to membrane bound cytochromes [90].

Both YcnJ and CopCD transporters tend to contain more charged and metal-binding amino acids in the resistant *Frankia* strains (not shown) and they form separate groups phylogenetically (Additional file 6). *Frankia sp.* strain EuI1c also has a third CopD (FraEuI1c_1869) which lacks the Caa3 domain, has increased metal binding amino acids, and groups phylogenetically with the two CopD proteins from the highly copper resistant *C. metallidurans* (Additional file 6). These two *copD* genes (Rmet_5668 and Rmet_6115) are up-regulated either with copper or with copper and other divalent cations (respectively) (Additional file 1).

The transcriptional response to copper was examined in *Frankia sp.* strain EuI1c [91]. All of the predicted copper transporters (CopD and YcnJ) and copper binding proteins (CopC and CopZ) are up-regulated in the presence of elevate copper levels [91]. In summary, duplication and modification of amino acids in copper transporters, CopD for strain EuI1c and CopA for strains DC12 and CN3, may explain the high resistance in these *Frankia* strains. Interestingly, these three copper-resistant strains are also atypical (non-nitrogen fixing) *Frankia* strains, although there is no clear link between nitrogen fixation and copper sensitivity.

### Molybdenum homeostasis

Molybdenum is found as part of an iron-sulfur cofactor in nitrogenase or combined with a tricyclic pterin forming the molybdenum cofactor (MoCo) [72]. MoCo oxidoreductases are nearly ubiquitous in life, and include many important enzymes classes including several dehydrogenases and ferredoxin oxidoreductases [92]. Molybdenum enters the cell through the high affinity ABC cassette (ModABC) or through low affinity sulfate permeases, SulT and SulP, due to its chemical similarity to sulfate [73]. All of the *Frankia* genomes have the ModABC cassette and several copies of SulP for high and low-affinity molybdate transport (Table 2). The molybdate chaperone (ModG) was only found in the genomes of nitrogen-fixing *Frankia* strains (Table 2), suggesting ModG is involved in the synthesis of the iron-molybdate nitrogenase cofactor [72]. The non-nitrogen-fixing *Frankia* genomes CN3, DC12, and EuI1c contain a sulfate export protein (TauZ). Since molybdate will enter through general sulfate importers, it is possible that TauZ could function to export surplus molybdate. All nitrogen-fixing *Frankia* strains lack TauZ, possibly to retain their molybdenum reserves.

### Selenium homeostasis

Selenium, in the form of selenocysteine or selenomethionine, is found in several stress proteins including glutathione peroxidase, alkyl hydroperoxidase, and multiple disulfide reductases [93]. The deprotonated electrons of selenium cofactors make the selenoproteins reduction-oxidation reactive, explaining why many identified selenoproteins are involved in thiol and oxidative stress resistance [93]. Since selenite generates these stresses in the cell, the stress-related selenoproteins may function in both detoxification and removal of free selenite ions from the cytoplasm [94]. Only 20% of sequenced bacteria are known to contain selenoproteins [47]. All of the *Frankia* genomes contained selenoproteins (Table 1).

As a sulfur analogue, selenium oxyanions enter cells through sulfate transporters such as SulP and CysPUWA, but selenite competitively inhibits the sulfate uptake through SulP [73]. In this study, four *Frankia* strains (DC12, EUN1f, EuI1c, and CN3) have elevated levels of selenite resistance (up to 3.5 mM) compared to *E. coli* K12 (0.5 mM) [5]. Only the genomes from these selenite-resistant *Frankia* strains contained both the SulP and CysPUWA sulfate permeases genes (Table 2), which are likely to prevent sulfur starvation in the presence of selenite.

Once inside the cell, selenite is reduced to inert insoluble elemental selenium, which has red color [95]. A red precipitate is formed by the selenite resistant *Frankia* strains CN3, EuI1c, EUN1f and DC12 implying selenite reduction [5]. Recently, selenite reduction and selenium nanosphere production was verified in *Frankia* strain EuI1c [96]. Small thiol-containing molecules, like glutathione, reduce selenite to seleno-diglutathione, which is further reduced to selenopersulfide by glutathione reductase [95]. Only *Frankia* EuI1c, EAN, and CN3 genomes contained glutathione synthase (GSH) (Table 2). However, all of the *Frankia* genomes contained synthase proteins for small thiols like mycothiol (MSH) (Table 2), which may substitute for glutathione for metal resistance [97]. Selenium is also reduced enzymatically either through thioredoxin and its reductase (TrxA and B) or nitrate/nitrite reductases [98]. These reductases are found in all *Frankia* genomes, likely to supply the cell with selenopersulfide. The selenopersulfide is either integrated into proteins via selenocysteine or spontaneously dismutates into elemental selenium [95]. The *Frankia* CN3 genome has a second type of nitrate reductase (NasC) that was associated with nitrite reductases (NirBD), and may contribute to the greater levels of selenite resistance in *Frankia* strain CN3 (Additional file 10).

Selenium-resistant strains also contained sulfite oxidase genes (FraEuI1c_6626, FrCN3DRAFT_7646, FrEUN1f_7579). Sulfite oxidase (SorA) converts selenite to the less toxic selenate [98, 99]. In summary, *Frankia* selenite resistance is likely due to alternate sulfate transporters (CysPUWA) that prevents sulfur starvation. Additional selenite resistance may result from oxidation of selenite to the less toxic selenate using SorA, or from selenite reduction. The selenite reduction observed in resistant strains could occur through several mechanism including mycothiol, TrxAB, YedY, or NasC/NirBD.

### Part 2: Toxic metals

#### Arsenate resistance

Being structurally analogous to phosphate, arsenate enters the cells through phosphate uptake transporters and acts as a metabolic poison [100]. Arsenate resistance involved the reduction of arsenate to arsenite, which is transported out of the cell [101]. Arsenate reduction to arsenite occurs by either the glutaredoxin-dependent ArsC (KO:K00537) or a thioredoxin-dependent phosphotyrosine-phosphatase (PTPase) termed ArsC2 (KO:K03741) [102]. These reductases are typically found in an *ars* operon with an *arsR* regulator and one of several types of arsenite transporters (*arsAB*, *arsB*, *acr3*, or *aqpS*) [100]. Arsenate reduction by *Frankia* likely involves ArsC2 and mycothiol, which substitutes for glutathione in the arsenate reduction process [102]. Glyoxalase I isomerizes hemithioacetal adducts (glutathione recycling), is responsive to various heavy metals, and is often found associated with *ars* operons in bacteria [103–105]. A cadmium-inducible glyoxyalase I (CadI) was also found within all of the potential *Frankia* arsenate resistance operons (data not shown). This evidence suggests CadI may assist in reducing glutathione/mycothiol-arsenical complexes.

Several *Frankia* strains are significantly more resistant to arsenate than *E. coli* (>50 mM vs. 10 mM), but not to arsenite (0.1 mM vs. 3 mM) [5]. A similar pattern of arsenate resistance and arsenite sensitivity was observed with *Sinorhizobium meliloti*, which has a novel pathway for arsenate resistance [106]. In *S. meliloti*, ArsC reduces arsenate to arsenite which is exported out of the cell via an aquaglyceroporin (AqpS) [106]. Only one (SMc02648) of the three AqpS homologs in *S. meliloti* is involved in arsenite export [106]. Most *Frankia* genomes contained at least two *aqpS* genes (Table 2). Phylogenetic analysis placed *Frankia* AqpS proteins on two of three distinct branches of aquaglyceroporins (Additional file 7). One branch of *Frankia* Aqps proteins was orthologous to SMc02648. The gene neighborhood of these *Frankia aqpS* genes contained the *arsR*, *arsC2* and *cadI* genes (not shown). *Frankia* EuI1c genome contained 2 copies of *aqpS* genes, but was 10-fold more sensitive to arsenate than other *Frankia* strain. The duplicated *aqpS* gene (FraEuI1c_6606) was missing the last two transmembrane domains, and had more positively-charged arginine residues (40 vs 9) on the long cytoplasmic c-terminal stretch. Possible explanations for this strain increased arsenate sensitivity is that this protein may form only a dysfunctional channel, or retain arsenic oxyanions on the positively charged cytoplasmic domains.

A second branch of the aquaporin (AqpZ) proteins contains two members that were up-regulated with heavy metals (Additional file 7). These AqpZ proteins were nearly identical to AqpS except they lacked the long cytoplasmic c-terminus (data not shown). The third branch of this phylogenetic tree contained homologs that were related to the glycerol uptake facilitators (GlpF), which are also known for arsenite-importing in both eukaryotes and prokaryotes [100]. No *glpF* genes were found in any of the *Frankia* genomes. Another arsenite transporter (ACR3), an arsenite sodium symporter, was identified in a few *Frankia* genomes (Table 2), but its presence did not correlate to the levels of arsenate or arsenite resistance.

### Chromate resistance

Chromate enters the cell through sulfate permeases, and is a potent inducer of oxidative stress and DNA repair mechanisms [107]. Chromate resistance involves export by the chromate permease (ChrA) followed by reduction either by ChrB or by a number of other proteins (nitrite reductase, quinone oxidoreductases, cytochromes, glutathione, etc.) [107, 108]. Most *Frankia* strains are more resistant to chromate than *E. coli*, *B. subtilis*, and *C. metallidurans*[5]. The presence or absence of ChrA in the *Frankia* genomes did not correlate to their levels of resistance. Other known chromate-resistance determinants [108–110] were either not present in the *Frankia* genomes or did not correlate to chromate resistance levels.

By looking at genes present only in more chromate resistant (MIC >1 mM) *Frankia* strains, 2 candidate chromate resistance genes were identified. One was a metallo-β-lactamase, which is unlikely to be involved in chromate resistance. The other gene coded for a possible metallothionein, which contained 3 or 4 CXXC motifs interspersed with multiple histidines (Additional file 9). Metallothioneins are involved in chromate binding and reduction through thioester bond formation [111], and are found in association with the ChrA transporter in related actinobacteria [112]. Therefore, it is possible that this metallothionein contributes to chromate resistance in *Frankia*.

### Lead resistance

Lead enters cells through Fe^2+^ and Ca^2+^ transporters, and exerts its toxicity by displacing these cations at their binding sites in metalloproteins [113]. The most well-characterized lead resistance mechanism occurs in *C. metallidurans*. This mechanism involves lead export through the Zn^2+^-type ATPase (PbrA), followed by precipitation on the cytoplasmic membrane through the combined action of a PAP-2 phosphatase and signal peptidase (PbrB and PbrC, respectively) [114]. Lead easily forms stable lead-phosphate precipitates. Several gram-positive and gram-negative bacteria exploit this property to detoxify lead as intra- or extracellular precipitates [115].

All of the *Frankia* strains are highly lead resistant with up to 8-fold higher levels compared to *C. metallidurans* and at least 2-fold greater than *S. viridochromogenes* levels [5]. *Frankia* was also able to bind significant amounts of lead as a precipitate [116]. In a genome-wide array study of *C. metallidurans* under lead stress, the most lead-responsive gene was the *copA*-like gene (Rmet_3524) [6]. The lead resistance genes, *pbrABCDT,* are up-regulated primarily with zinc and cadmium stress with only *pbrA* being up-regulated with lead stress [6]. All of the *Frankia* genomes contained *copA* genes and these gene products were significantly divergent from the other characterized CopA proteins (Additional file 5). One possibility is that the *Frankia* Cu^2+^-ATPases also export other metals like Pb^2+^. Phosphate utilization (e.g. polyphosphate phosphatase) and cell surface modification proteins (e.g. UDP-glucose 4-epimerase) were located within the highly conserved gene neighborhood of the *Frankia copA* genes (Additional file 8). These proteins are known to play a role in the surface binding of Pb^2+^[117].

In a *Bacillus* isolate which lacked the *pbrA* and *pbrT* genes, lead was exported by alternate transporters, but is still bound extracellularly [118]. Among the genes that are up-regulated with lead stress, there are 3 paralogous operons containing the genes for undecaprenyl phosphatase (UppP) and DUF347 protein (Additional file 1). Although UppP does not necessarily cause metal resistance, it may help to stop the futile metal import-export cycle by precipitating metals on the cell surface [119]. The DUF347 protein is 6 trans-membrane-segment protein that may work in conjunction with the co-expressed UppP as another metal export-precipitation system. In *Thalassiosira pseudonana*, *DUF347* is the most highly up-regulated gene that was divalent metal-specific [120] supporting this hypothesis. All of the *Frankia* genomes contained at least one copy of DUF347 and multiple copies of UppP (Table 2). In contrast, only 3 of the 18 non-*Frankia* bacterial genomes contained a DUF347 or multiple UppP orthologs (Additional file 10). The presence of DUF347 or multiple UppP orthologs could explain the high lead resistance levels for these *Frankia* strains.

### Tellurite resistance

Tellurite is considered a biologically useless metalloid. Due to its induction of oxidative stress in bacteria, tellurite is toxic at micromolar levels [121]. Tellurite enters bacterial cells through either the phosphate transporter (PitA) or nitrate transporters [122, 123]. Similar to selenite, tellurite is detoxified through a reduction mechanism by nitrate/nitrate reductases and small cellular thiols like glutathione, leaving an insoluble black precipitate [121, 124]. Additionally, there are specific tellurite resistance genes including *telAB*, *terABCDEZ*, and *tehAB*, but little information exists on their exact functions [121]. While tellurite resistance levels have not been established for *Frankia*, there were several factors that suggested potential tellurite resistance. Many *Frankia* strains were also resistant to other metal oxyanions like selenite and arsenate. Secondly, all of the *Frankia* genomes had a full set of *terABCDEZ* genes and nitrite reductases (NirBD). Bacteria having *terABCDEZ* operons are frequently resistant up to 4 mM tellurite [125]. *Frankia* CcI3 and CN3 genomes also had nitrate reductase (NasC) gene, which could be used for selenite or tellurite reduction [121]. To test our hypothesis and to distinguish between tellurite and selenite resistance, *Frankia* strain CcI3, which is selenite-sensitive, was grown on tellurite agar. *Frankia* strain CcI3 was able to grow in the presence of tellurite and produced black elemental tellurium in medium containing up to 3 mM TeO_3_ (Additional file 19).

### Metal regulation

The MerR family contains several types of self-regulating transcriptional activators of genes involved in copper/silver resistance (CueR), gold (GolS), mercury resistance (MerR), zinc (ZntR), cadmium (CadR), lead (PbrR), cobalt (CoaR), oxidative stress (SoxR), nitrogen metabolism (GlnR), biofilm formation (MlrA), and heat shock (DnaK) [126–128]. There are between 9 and 20 MerR proteins in *Frankia* genomes (Additional file 10), which phylogenically cluster into ten groups (Additional file 20). Four of these nine groups could be metal responsive based on a conserved amino acid motif (Additional file 10) derived from sequence alignments of metal induced MerR genes from compiled gene arrays (Additional file 1). None of the metal-related *merR* genes are located in operons with metal resistance proteins, so are likely regulating acting *in trans*.

The second largest family of metal regulators in *Frankia* include members of ArsR/SmtB-family. Originally characterized as a repressor of the arsenic responsive *ars* operon, members of this family can respond to a variety of metals (nickel, zinc, copper, cadmium, lead), causing de-repression of regulatory targets [129]. In *Frankia*, ArsR proteins phylogenetically cluster into 5 groups. One groups is part of the potential arsenic resistance operon (Additional file 21). There is also a large group of ArsR-like proteins in a potential operon with activator of HSP90 ATPases (AHSA1), though a direct link between ArsR and AHSA1 expression has not been elucidated. A third group shows sequence similarity to the nickel sensor from *M. tuberculosis* (NmtR), though retain no synteny or proximity to metal transporters. A small group of ArsR proteins are orthologous with the cadmium and lead responsive CmtR of *M. tuberculosis* and nickel responsive SrnR of *Streptomyces griseus*[130, 131]. Members of this group of *Frankia* ArsR proteins are in a possible operon with the metal exporter CzcD (Additional file 21). The last group shows no homology to any characterized ArsR family proteins and are not proximal to other metal related genes.

Another family of transcriptional repressors includes orthologs of the copper-sensitive operon repressor (CsoR) [132]. These regulators are primarily responsive to either copper (CsoR, RicR) or to nickel and cobalt (DmeR, RcnR), though other metals have been demonstrated to induce gene derepression by these regulators [132–136]. Several CsoR proteins were demonstrated to regulate metal exporters such as Cu-ATPase (CopA) [133]. *Frankia* contains orthologs to CsoR, RicR, and an uncharacterized CsoR-like protein (Additional file 22). The *Frankia* sp. strain CN3 CsoR ortholog (FrCN3DRAFT_2771) is directly downstream of CopA, maintaining synteny with characterized CsoR proteins (Additional file 22).

The other *Frankia* strains contain orthologs to RicR_Mtub_ (Additional file 22), which is responsive to copper, iron, and zinc [134]. A branch of *Frankia* CsoR-like proteins (Franean1_3360, FrEUN1f_0873, FrCN3DRAFT_5236) cluster with an uncharacterized regulator (Rv1766) and to a lesser extent the copper and nickel responsive CsoR_Mtub_ (Additional file 22). These CsoR-like proteins have the amino acid motif (R..R…Q......M.[ED]…[DE]C.....Q.{22}[HC]) of nickel regulators RcnR/DmeR, which lacks the tyrosine (Y) and H…C section of the CsoR/RicR motif (R.{13}M…..YC.D.*H…C). Only the *Frankia* genomes containing an ortholog to nickel-exporting RcnA (Additional file 10) have orthologs of the uncharacterized CsoR-like protein, further suggesting that they may be involved in nickel regulation (Additional file 22).

The iron transport repressor (Fur) binds a variety of essential divalent metals including iron (Fur), zinc (Zur), nickel (Nur), manganese (Mur), or cobalt (Co-Fur) the combination of which determines the level of Fur transcription [137]. Bacteria contain an additional layer of Fur-family proteins (eg. PerR) which also bind metals and regulate the transcription of Fur [137]. Orthologs of Fur and Nur have been previously examined in *Frankia* which retain a high level of protein sequence conservation to characterized proteins [138, 139]. In *Frankia*, Fur is located in an operon with either KatA or KatG catalases (Additional file 23), which rely on iron (heme) to function [140, 141]. There are two phylogenetic groups of *Frankia* Nur proteins (Additional file 23). For cluster I *Frankia nur* is located adjacent to a metallopeptidase (*gcp*) and the nickel chaperone *ureG*. In cluster III and IV *Frankia* genomes, *nur* is upstream of an iron-sulphur cluster repair protein (YgfZ). The characterized *S. coelicolor* Nur ortholog is also co-located with YgfZ and has been demonstrated to regulate nickel import genes (*nikABCDE*) and oxidative stress (*sodF*) [139].

Another Fur paralog (Zur) regulates zinc importers, and is itself regulated by ArsR as demonstrated in *M. tuberculosis*[142]. ArsR is zinc responsive and may work with Zur to regulate intracellular zinc through repression and derepression of the cation transporter CzcD [143]. While all *Frankia* genomes contained a Zur regional to the zinc importer ZntABC, *Frankia* sp. strain DC12 has a second Zur in an operon with ArsR (Additional file 23). The remaining Fur protein in *Frankia* (PerR) is in operon with rubrerythrin, which binds iron and zinc and is involved in oxidative stress response [144]. The *Frankia* genomes contain a second type of iron regulatory protein, diptheria toxin repressor (DtxR) (Additional file 10), which regulate several iron related proteins including siderophores and iron storage proteins [145]. The *Frankia* DtxR proteins are distinguished from the closely related manganese regulator (MntR) by the conservation of four amino acid residues previously identified as iron binding [146].

## Conclusions

*Frankia* has successfully adapted to a challenging habitat: an acidic rhizosphere full of secondary metabolites, plant phenolics, and solubilized metals. To accomplish this feat, *Frankia* has a full complement of proteins that use all major beneficial metals. *Frankia* metal resistance patterns appear to be unique to each strain. The high levels of copper resistance appears to be due to duplicated *copD* genes in *Frankia sp.* strain EuI1c and unusual *copA* genes in strains CN3 and DC12. Selenite resistance involves an alternate sulfur importer (CysPUWA) working with reductases to produce elemental selenium. Nickel, cobalt, zinc, and cadmium resistance are likely the result of a combination of CDF proteins. Lead appears to be exported via Cu^2+^- ATPases or DUF347, and precipitated as a bound metal phosphate similar to other bacterial species. *Frankia* arsenate resistance appears to be achieved through a novel mechanism utilizing an aquaporin (AqpS) that was discovered in nitrogen-fixing *S. meliloti*. The *terABCDEZ* genes and various reductases provide *Frankia* CcI3 (and possibly other *Frankia* strains) with tellurite resistance. *Frankia* has achieved similar levels of metal and metalloid resistance as bacteria from highly metal-contaminated sites. The potential for use of *Frankia*-actinorhizal symbiosis in bioremediation efforts makes further research into expression of these genes worthwhile.

## Methods

### Gene identification in *Frankia*

An initial list of genes involved in *Frankia* metal homeostasis was created using a combination of comparative analyses, motif searches, PERL algorithms, and microarray data (Additional file 24). Comparative analysis of *Frankia* and other genomes was performed primarily using the Integrated Microbial Genome (IMG) database [147]. Metal homeostasis genes identified in the literature that lacked locus tags or gene accession numbers were identified through the UniProt database [148]. Orthologs of genes from functionally characterized proteins of other microorganisms were identified in *Frankia* using the embedded BLASTp software on IMG. Proteins with a minimum of 30% identity and e-values <10^−20^ were considered orthologs.

### Low scoring and hypothetical proteins

The large phylogenetic distance between *Frankia* and model organisms, such as *E. coli* and *B. subtilis*, meant many of the closest metal transport homologs in *Frankia* had less than 30% identity and e-values of >10^−20^. These low-scoring genes, as well as all hypothetical proteins were: 1. examined for conservation of domains from other databases including protein families (Pfam) [149], gene ontology [150], and cluster of orthologous groups (COG) [151], 2. individually analyzed for conserved metal binding residues as compared to characterized metal resistance genes, 3. visually checked for neighborhood synteny and clustering with other metal homeostasis genes using native IMG tools [147].

### Gene array meta-analysis

Genes from 16 different published whole genome array studies involving bacterial response to heavy metals were analyzed as follows. Any potential metal homeostasis proteins that were significantly (based on originating paper cut-off) up-regulated were included for analysis. Genes were assigned an IMG identifier by downloading the genome data from IMG for each organism and matching genes from the arrays to the IMG data using Excel tools. The genes were uploaded to IMG and the various information about the genes (symbol, COG numbers, protein domains…etc) were obtained and exported. The expression data were matched to the downloaded IMG gene information and this collective data was combined and analyzed in Excel (Additional file 1).

To normalize gene expression between studies, the genes of each study were ranked by dividing the fold change of each gene by the maximum fold change in that study. Thus, the gene with the maximum response has a value of 1 and the remaining genes are a fraction of the maximal response (Additional file 1). Up-regulated genes in multiple different species/array studies were selected for analysis. BLASTp searches were done using IMG to identify orthologs of these genes in the *Frankia* genomes.

### Assessment of metal binding potential

Using the regular expression search function on IMG and in-house PERL algorithms, the *Frankia* genome were searched for proteins containing either conserved metal binding motifs obtained from the literature or high metal-binding potential as previously described [112]. Since each genome has a unique amino acid distribution, all proteins in each genome were scaled by % of each individual amino acid. Proteins in the top 1% highest cysteine or histidine were considered potentially metal binding and examined for other metal homeostasis factors, including: neighboring metal-related genes, presence/absence in metal resistant strains, orthology to genes up-regulated with metal in array studies.

### Phylogenic tests of relatedness

To distinguish highly similar mechanisms or metal specificity, phylogenetic trees were constructed with proteins from *Frankia* and organisms that were either: 1. from metal homeostasis literature, 2. from past studies involving *Frankia* metal resistance, and/or 3. from the compiled gene array studies. General comparative organisms for gene identification can be found in Additional file 10 and include either organisms used in the Richards et. al. 2002 analysis of *Frankia* metal resistance or organisms with model metal homeostasis systems in 4 major categories: actinobacteria, Gram positive and negative bacteria, and eukaryotes (to demonstrate genetic distance). Alignments were created using ClustalW in BioEdit [152], and neighbor-joining trees with 1000 bootstraps were constructed using MEGA software [153]. Functionality or metal specificity was tentatively assigned based on grouping with experimentally characterized proteins from the literature.

### Ethics

This study did not involve humans, animals or plants in any manner.

## Electronic supplementary material

Additional file 1: **Compiled data from global gene expression studies in response to metals.** Genes from 16 global gene expression studies were identified in the IMG database. Protein data was downloaded, matched to expression levels, and organized in Excel. To be able to compare across studies, the gene with the maximal expression in each study was set to 1 with all other genes normalized to that gene. COG and Pfam counts were also calculated and genes with high COG or Pfam counts that were also found in multiple organisms per metal were used to: 1. identify novel resistance mechanisms, 2. confirm likeliness of identified resistance mechanisms in *Frankia*, and 3. determine metal specificity of identified mechanisms (if any). (XLS 2 MB)

Additional file 2: **Phylogeny of** ***Frankia*** **iron-siderophore ABC cassettes.** Un-rooted neighbor-joining tree of iron ABC permeases in *Frankia*, those characterized in *E. coli*, and those up-regulated with heavy metals (underlined) (Additional file 1). Identifying COG domains found in the displayed proteins are shown on their respective branches. (PPT 576 KB)

Additional file 3: **Phylogeny of** ***Frankia*** **metal chaperones containing COG0523 domains.** Circular neighbor-joining tree of *Frankia* proteins identified by BLASTp as orthologs of experimentally characterized (underlined) members of COG0523 family metal chaperones. Orthologs from metal resistant *C. metallidurans* CH34, *Streptomyces viridochromogenes* DSM 40736, *Bacillus subtilis subtilis* 168, *Escherichia coli* K12- W3110, and *Helicobacter pylori* B8 are shown for comparison. Characteristically defining domains for nickel cofactored HypB (KO:K04652) and UreG (KO:K03189), and the cobalt cofactored CobW (KOG2743) are shown on their associated branches. (PPT 634 KB)

Additional file 4: **Nickel import vs export permeases.** Neighbor-joining tree of *Frankia* NixA-family permeases similar to either A. the nickel exporting RcnA (COG2215) or B. the nickel importing NhlF (KO:K07241). Proteins containing either domain that were up-regulated in the compiled array studies were also included. *E. coli* homolog to RcnA is YohM (Missing locus_tag). (PPT 104 KB)

Additional file 5: **Phylogeny of Frankia Cu** ^**2+**^ **-ATPase proteins.** Neighbor-joining tree of ClustalW aligned CopA proteins sequences from *Frankia*, *E. coli*, *S. viridochromogenes* and all Zn^2+^- or Cu^2+^- type ATPases up-regulated in the compiled array studies (underlined). *Frankia* CopA proteins (B) form a distinct group from characterized CopA proteins (A) in other bacterial species. The exceptions (C) are FrCN3DRAFT_2755 and FraDC12DRAFT_3523 which are highly homologous to the predicted copper resistance gene CopF of *Cupravideous metallidurans* (Rmet_6119). Of the *Frankia* genomes analyzed, only *Frankia sp.* strain DC12 has a ZntA type ATPase (D) though both CopA and ZntA proteins are up-regulated with a variety of metals (shown on their respective branches). *E. coli* K-12 W3110 CopA and ZntA is indicated with its gene name in black, all others are locus tags. * = DRAFT, ** = 0101000. (PPT 497 KB)

Additional file 6: **Phylogeny of** ***Frankia*** **CopC and CopD proteins.** Neighbor-joining tree of ClustalW aligned CopC and CopD protein sequences from *Frankia* and comparative organisms used in Richards et. al. 2002. Genes up-regulated with metals in compiled gene array studies are underlined. Identifying Pfam domains for each group of proteins was also included to show characteristics of *Frankia* CopCD proteins. * = DRAFT, ** = 0101000. (PPT 536 KB)

Additional file 7: **Phylogeny of** ***Frankia*** **arsenic transport permeases.** Neighbor-joining tree of ClustalW aligned major intrinsic protein (MIP) domain (pfam00230) containing protein sequences from *Frankia*, from compiled gene arrays (underlined), and from several experimentally characterized genes. The novel arsenite exporting aquaporin from *Sinorhizobium meliloti* is in bold. *Frankia sp.* strain EuI1c contains a unique MIP (A) which may contribute to its sensitivity to arsenate. * = DRAFT, ** = 0101000. (PPT 791 KB)

Additional file 8: **Gene neighborhood synteny of** ***Frankia*** **-type Cu** ^**2+**^ **-ATPases.** The region contains genes for electron transport chain components [heme B, cytochrome C, ubiquinone (CoQ)/menaquinone (VitK)] and copper sequestration to the cell surface [phosphate metabolism and cell surface modification genes]. Association of CopA with this region suggests a dual purpose of decreasing heme disruption by cytoplasmic copper and delivering copper to the ETC complex IV. CopA in *Frankia* strains EAN, EUN, and CopZA in DC12 have been transposed to a different region in these genomes. (PPT 97 KB)

Additional file 9: **Protein alignment of a potential metallothionein involved in chromate resistance.** ClustalW alignment of a metallothionein found only in the chromate resistant bacterial strains based Richards et. al. 2002. Brackets indicate potential metal binding motifs. Amino acids are colored by characteristic: dark blue are negatively charged (D/E), purple are uncharged metal-binding (C/H), red are positively charged (K/R), pink are cyclic (P), orange are metal-binding and hydrophobic (M), yellow are hydrophobic (L/I/V/M/G/A), green are aromatic (F/W/Y), light blue are polar (S/T/N/Q). (PPT 80 KB)

Additional file 10: **Summary of experimental gene data and evidence table.** Literature summary, array data summary, and metal binding motifs used to identify metal resistance mechanisms in *Frankia*. My symbol assigned to a *Frankia* protein is based on either the phylogenetically closest or most well characterized protein ortholog. An identifying domain to distinguish closely related mechanisms, as well as some of the conserved amino acid motifs, was set using combined blast and phylogeny in the IMG site and related databases. Colorized gene counts from all array and comparative [5] organisms along with a few well characterized organisms including *Helicobacter pylori*, *Streptomyces coelicolor*, *Staphylococcus aureus*, and *Saccharomyces cerevisiae* are included for visual comparison to evidence. (XLSX 41 KB)

Additional file 11: ***Frankia sp.*** **strain CcI3 metal homeostasis mechanisms.** Schematic diagram of known and putative metal homeostasis systems in *Frankia* CcI3. Loci containing identifying domains (see Additional file 10) for metal ion uptake transporters, chaperones, modification enzymes, efflux transporters, and surface binding protein and efflux systems are shown (left to right) with arrows to indicate the flow of metals through the cell. Information at the bottom indicates whether the strain is symbiotic with host plants (Sym^+/-^), is a diazotroph (N_2_-fix^+/-^), and whether the strain is resistant (r) or sensitive (s) to a particular metal. (PPT 184 KB)

Additional file 12: ***Frankia sp.*** **strain QA3 metal homeostasis mechanisms.** Schematic diagram of known and putative metal homeostasis systems in *Frankia sp.* strain QA3. Loci containing identifying domains (see Additional file 10) for metal ion uptake transporters, chaperones, modification enzymes, efflux transporters, and surface binding protein and efflux systems are shown (left to right) with arrows to indicate the flow of metals through the cell. Information at the bottom indicates whether the strain is symbiotic with host plants (Sym^+/-^), is a diazotroph (N_2_-fix^+/-^), and whether the strain is resistant (r) or sensitive (s) to a particular metal. * = DRAFT. (PPT 184 KB)

Additional file 13: ***Frankia*** **alni ACN14a metal homeostasis mechanisms.** Schematic diagram of known and putative metal homeostasis systems in *Frankia* alni ACN14a. Loci containing identifying domains (see Additional file 10) for metal ion uptake transporters, chaperones, modification enzymes, efflux transporters, and surface binding protein and efflux systems are shown (left to right) with arrows to indicate the flow of metals through the cell. Information at the bottom indicates whether the strain is symbiotic with host plants (Sym^+/-^), is a diazotroph (N_2_-fix^+/-^), and whether the strain is resistant (r) or sensitive (s) to a particular metal. (PPT 183 KB)

Additional file 14: ***Frankia sp.*** **strain EUN1f metal homeostasis mechanisms.** Schematic diagram of known and putative metal homeostasis systems in *Frankia sp.* strain EUN. Loci containing identifying domains (see Additional file 10) for metal ion uptake transporters, chaperones, modification enzymes, efflux transporters, and surface binding protein and efflux systems are shown (left to right) with arrows to indicate the flow of metals through the cell. Information at the bottom indicates whether the strain is symbiotic with host plants (Sym^+/-^), is a diazotroph (N_2_-fix^+/-^), and whether the strain is resistant (r) or sensitive (s) to a particular metal. (PPT 185 KB)

Additional file 15: ***Frankia sp.*** **strain EAN1pec metal homeostasis mechanisms.** Schematic diagram of known and putative metal homeostasis systems in *Frankia sp.* strain EAN1pec. Loci containing identifying domains (see Additional file 10) for metal ion uptake transporters, chaperones, modification enzymes, efflux transporters, and surface binding protein and efflux systems are shown (left to right) with arrows to indicate the flow of metals through the cell. Information at the bottom indicates whether the strain is symbiotic with host plants (Sym^+/-^), is a diazotroph (N_2_-fix^+/-^), and whether the strain is resistant (r) or sensitive (s) to a particular metal. (PPT 192 KB)

Additional file 16: ***Frankia sp.*** **strain EuI1c metal homeostasis mechanisms.** Schematic diagram of known and putative metal homeostasis systems in *Frankia sp.* strain EuI1c. Loci containing identifying domains (see Additional file 10) for metal ion uptake transporters, chaperones, modification enzymes, efflux transporters, and surface binding protein and efflux systems are shown (left to right) with arrows to indicate the flow of metals through the cell. Information at the bottom indicates whether the strain is symbiotic with host plants (Sym^+/-^), is a diazotroph (N_2_-fix^+/-^), and whether the strain is resistant (r) or sensitive (s) to a particular metal. (PPT 185 KB)

Additional file 17: ***Frankia sp.*** **strain CN3 metal homeostasis mechanisms.** Schematic diagram of known and putative metal homeostasis systems in *Frankia sp.* strain CN3. Loci containing identifying domains (see Additional file 10) for metal ion uptake transporters, chaperones, modification enzymes, efflux transporters, and surface binding protein and efflux systems are shown (left to right) with arrows to indicate the flow of metals through the cell. Information at the bottom indicates whether the strain is symbiotic with host plants (Sym^+/-^), is a diazotroph (N_2_-fix^+/-^), and whether the strain is resistant (r) or sensitive (s) to a particular metal. * = DRAFT. (PPT 201 KB)

Additional file 18: ***Frankia sp.*** **strain DC12 metal homeostasis mechanisms.** Schematic diagram of known and putative metal homeostasis systems in *Frankia sp.* strain DC12. Loci containing identifying domains (see Additional file 10) for metal ion uptake transporters, chaperones, modification enzymes, efflux transporters, and surface binding protein and efflux systems are shown (left to right) with arrows to indicate the flow of metals through the cell. Information at the bottom indicates whether the strain is symbiotic with host plants (Sym^+/-^), is a diazotroph (N_2_-fix^+/-^), and whether the strain is resistant (r) or sensitive (s) to a particular metal. * = DRAFT. (PPT 182 KB)

Additional file 19: **Confirmation of** ***Frankia*** **resistance and reduction of tellurite based on genome prediction.** *Frankia* sp. strain CcI3 was grown on solid minimal media containing either [A] 0 mM or [B] 3 mM potassium tellurite. As predicted by the presence of several tellurite resistance and reduction factors in the *Frankia* genome, strain CcI3 was able to grow in the presence of tellurite and reduce it to elemental tellurium (black precipitate). (TIFF 4 MB)

Additional file 20: **Phylogenetic analysis of the MerR family proteins in** ***Frankia.*** Neighbor-joining tree of Clustal Ω aligned MerR proteins (COG0789) containing protein sequences from *Frankia*, *Bacillus subtilis subtilis* 168, *Escherichia coli* K12-W3110, and *Cupriavidus metallidurans* CH34. The ten clusters of *Frankia* MerR proteins comprise two distinct group of metal and non-metal regulators. Underlined genes were upregulated with the indicated metals from the compiled gene array studies (see Additional file 1). Asterisk indicates the conservation of a motif. ([LIV][SADG][DER].....[DEGA].{3,4}[^S].[LIV][DR][FHDCY]Y.{3,4}GL[LIVMF].*[^Q][GR].[FY]..[^H]) derived from protein sequence alignment of the upregulated MerR array genes. (PPTX 406 KB)

Additional file 21: **Phylogenetic analysis of the ArsR family proteins in** ***Frankia.*** Neighbor-joining tree of Clustal Ω aligned ArsR proteins (COG0640) containing protein sequences from *Frankia*, upregulated genes from the compiled array data (Additional file 1), and characterized ArsR-family proteins. The metals that induced up-regulation of the gene from the array studies are listed next to the gene. The five clusters of *Frankia* ArsR proteins include 1. a group with no similarity to characterized ArsR proteins, 2. a group that is in a potential operon with the cation diffusion facilitor (CzcD), 3. a group that is in a potential operon with the activator of heat shock 90 ATPase protein (AHSA), 4. a group highly similar to the M. tuberculosis nickel responsive repressor (NmtR), and 5. a group in a potential *ars* operon with other arsenic resistance genes. (PPTX 387 KB)

Additional file 22: **Phylogenetic analysis of the CsoR family proteins in** ***Frankia.*** Neighbor-joining tree of Clustal Ω aligned CsoR proteins (pfam02583) containing protein sequences from *Frankia*, characterized members of CsoR-family, and proteins from the 18 comparative organisms (Additional file 10). 1. Uncharacterized CsoR-like proteins. 2. True CsoR proteins. The synteny between characterized CsoR (BSU33420) and the other orthologs is displayed, showing the regulatory target, copper-exporting CopA. 3. Copper regulating RicR proteins. (PPTX 689 KB)

Additional file 23: **Phylogenetic analysis of the Fur family proteins in** ***Frankia.*** Neighbor-joining tree of Clustal Ω aligned MerR proteins (KO:K03711) containing protein sequences from *Frankia*, and the 18 comparative organisms (Additional file 10). Representative neighborhoods are included to demonstrate synteny of the 4 regulators (Fur, Nur, PerR, Zur) in *Frankia*. Arrows indicate the regulators and their targets. Asterisks indicate characterized proteins. (PPTX 874 KB)

Additional file 24: **Methodology workflow chart.** Breakdown of the process for identification of metal homeostasis mechanisms used in this study. (PPT 61 KB)
