# Supplementary figures and images for: The ins and outs of metal homeostasis by the root nodule actinobacterium Frankia
Source: BMC Genomics. 2014 Dec 12;15:1092. doi: 10.1186/1471-2164-15-1092 (PMC4531530; doi:10.1186/1471-2164-15-1092)

## Slide 1
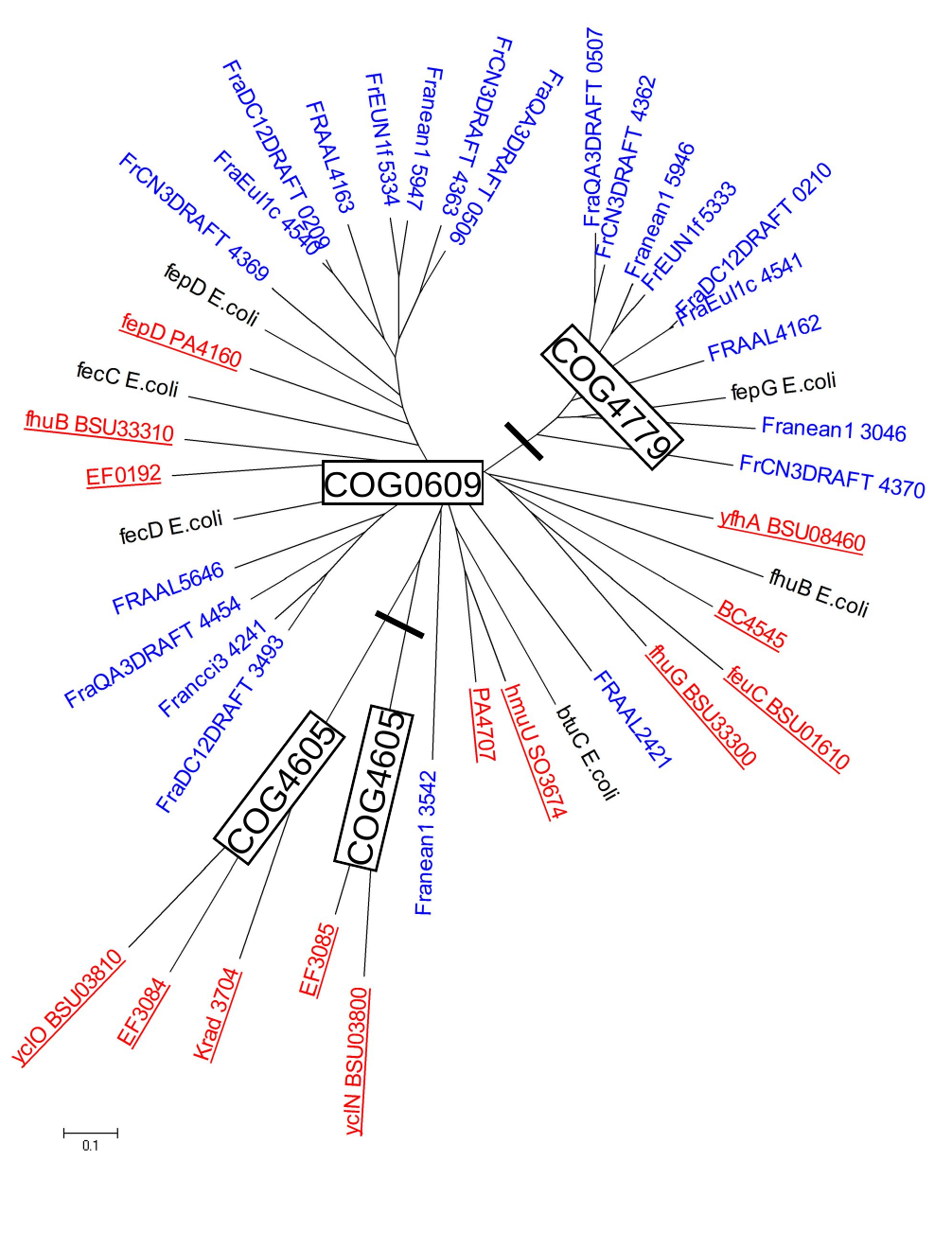

COG4779
COG0609
COG4605
COG4605

Supplement: Supplementary file 2 — Additional file 2: Phylogeny of Frankia iron-siderophore ABC cassettes. Un-rooted neighbor-joining tree of iron ABC permeases in Frankia, those characterized in E. coli, and those up-regulated with heavy metals (underlined) (Additional file 1). Identifying COG domains found in the displayed proteins are shown on their respective branches. (PPT 576 KB) [file 12864_2014_7073_MOESM2_ESM.ppt]

## Slide 1
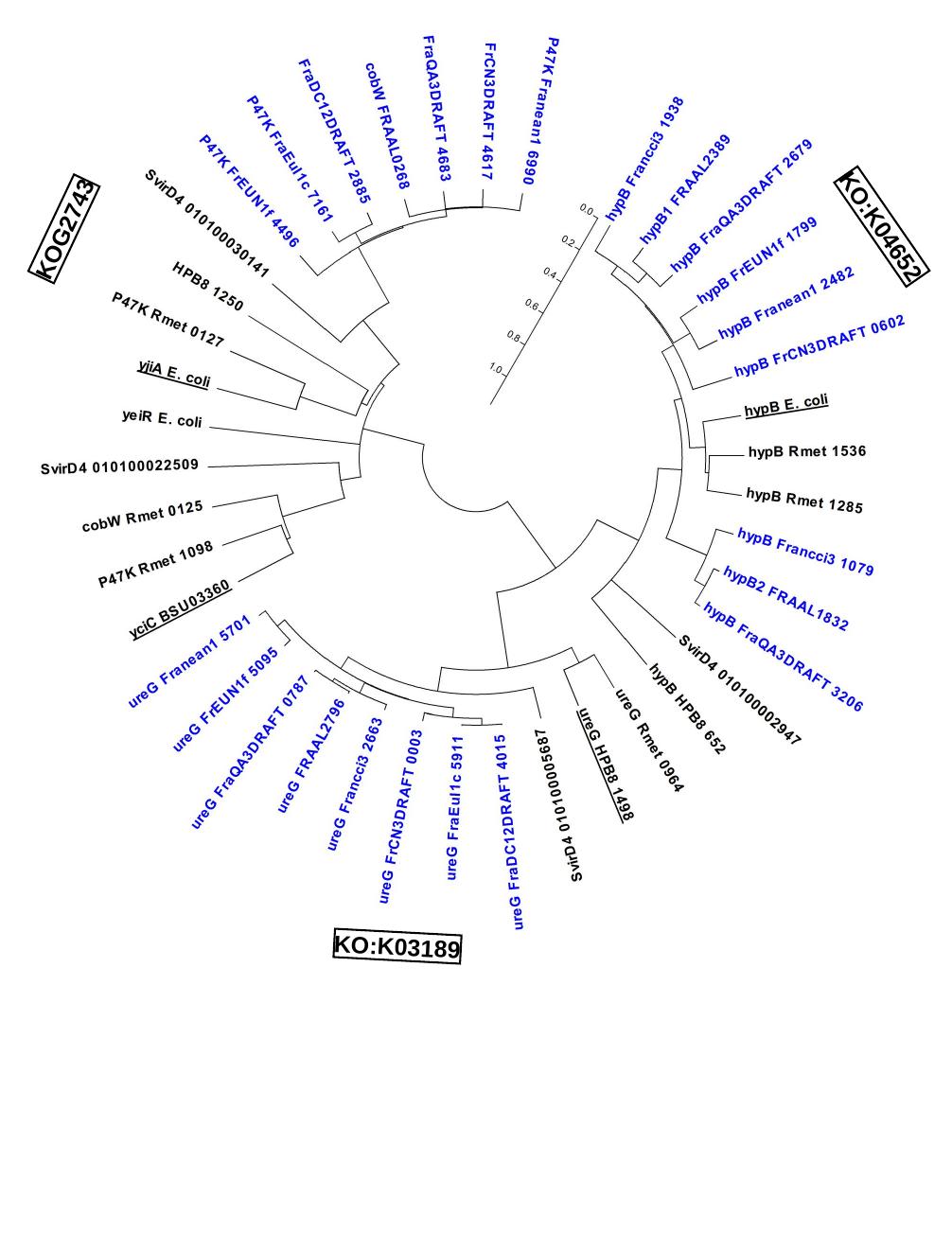

KO:K04652
KOG2743
KO:K03189

Supplement: Supplementary file 3 — Additional file 3: Phylogeny of Frankia metal chaperones containing COG0523 domains. Circular neighbor-joining tree of Frankia proteins identified by BLASTp as orthologs of experimentally characterized (underlined) members of COG0523 family metal chaperones. Orthologs from metal resistant C. metallidurans CH34, Streptomyces viridochromogenes DSM 40736, Bacillus subtilis subtilis 168, Escherichia coli K12- W3110, and Helicobacter pylori B8 are shown for comparison. Characteristically defining domains for nickel cofactored HypB (KO:K04652) and UreG (KO:K03189), and the cobalt cofactored CobW (KOG2743) are shown on their associated branches. (PPT 634 KB) [file 12864_2014_7073_MOESM3_ESM.ppt]

## Slide 1
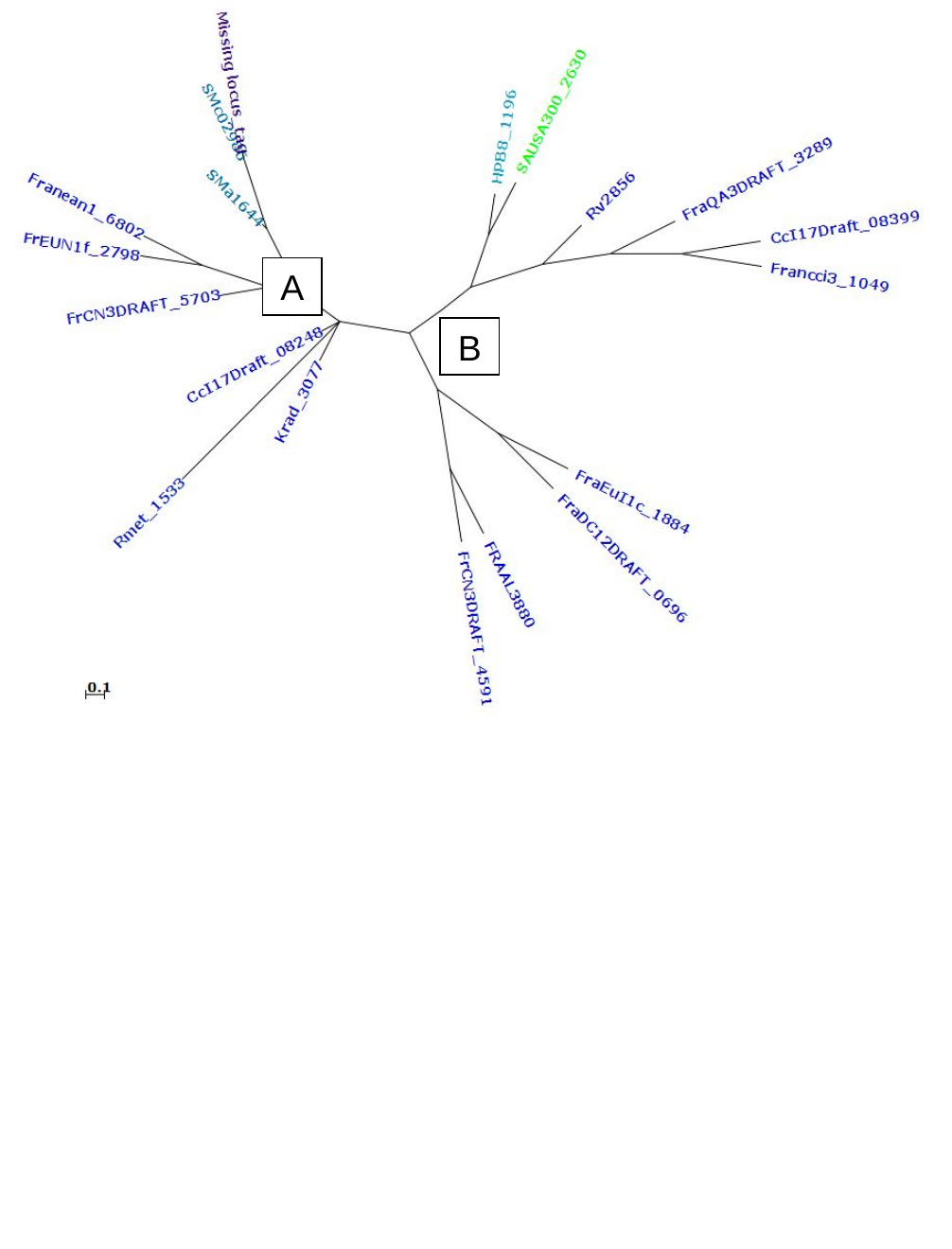

A
B

Supplement: Supplementary file 4 — Additional file 4: Nickel import vs export permeases. Neighbor-joining tree of Frankia NixA-family permeases similar to either A. the nickel exporting RcnA (COG2215) or B. the nickel importing NhlF (KO:K07241). Proteins containing either domain that were up-regulated in the compiled array studies were also included. E. coli homolog to RcnA is YohM (Missing locus_tag). (PPT 104 KB) [file 12864_2014_7073_MOESM4_ESM.ppt]

## Slide 1
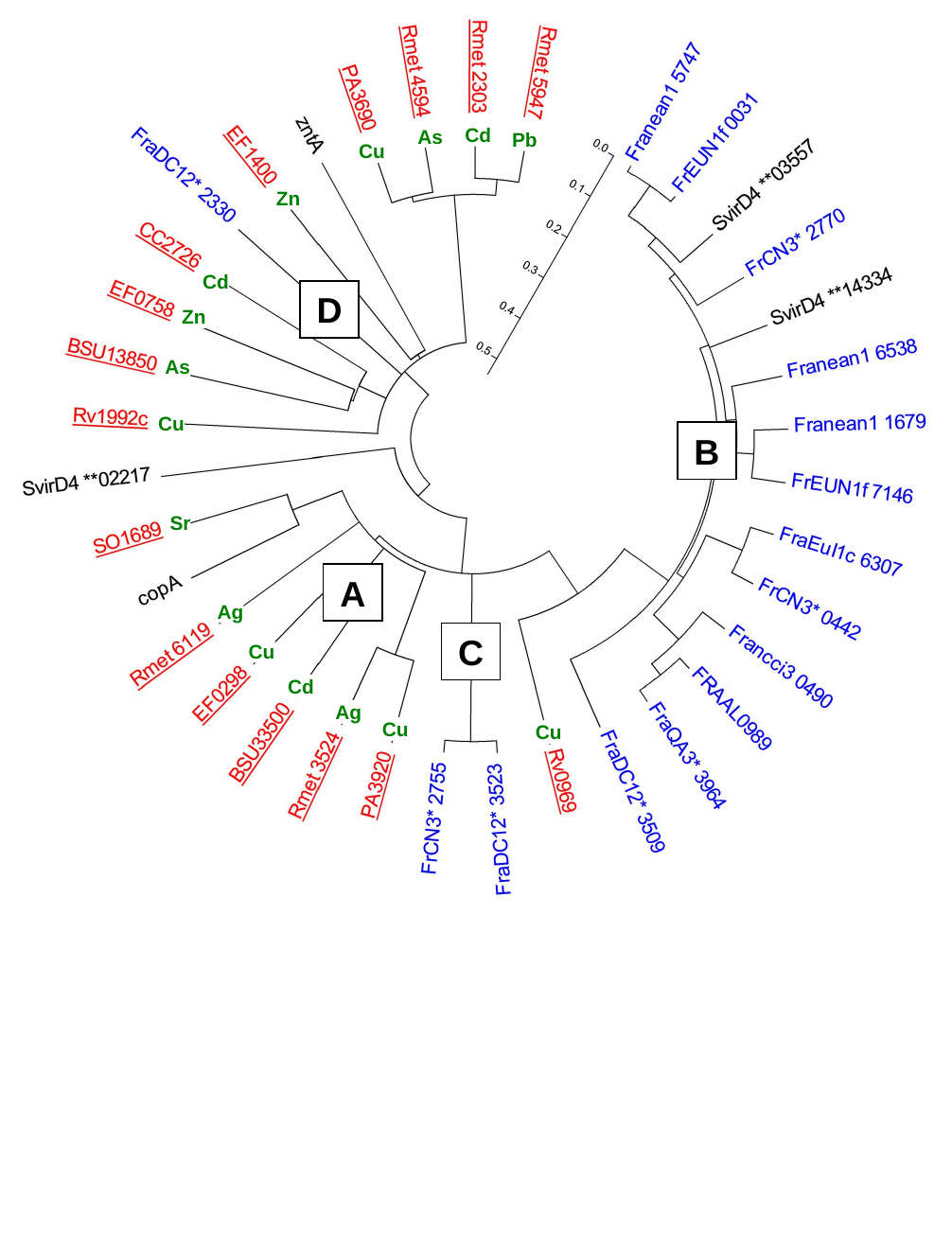

Cd
As
Pb
Cu
Zn
Cd
Zn
As
Cu
Sr
Ag
Cu
Cd
Ag
Cu
Cu
D
B
A
C

Supplement: Supplementary file 5 — Additional file 5: Phylogeny of Frankia Cu 2+ -ATPase proteins. Neighbor-joining tree of ClustalW aligned CopA proteins sequences from Frankia, E. coli, S. viridochromogenes and all Zn2+- or Cu2+- type ATPases up-regulated in the compiled array studies (underlined). Frankia CopA proteins (B) form a distinct group from characterized CopA proteins (A) in other bacterial species. The exceptions (C) are FrCN3DRAFT_2755 and FraDC12DRAFT_3523 which are highly homologous to the predicted copper resistance gene CopF of Cupravideous metallidurans (Rmet_6119). Of the Frankia genomes analyzed, only Frankia sp. strain DC12 has a ZntA type ATPase (D) though both CopA and ZntA proteins are up-regulated with a variety of metals (shown on their respective branches). E. coli K-12 W3110 CopA and ZntA is indicated with its gene name in black, all others are locus tags. * = DRAFT, ** = 0101000. (PPT 497 KB) [file 12864_2014_7073_MOESM5_ESM.ppt]

## Slide 1
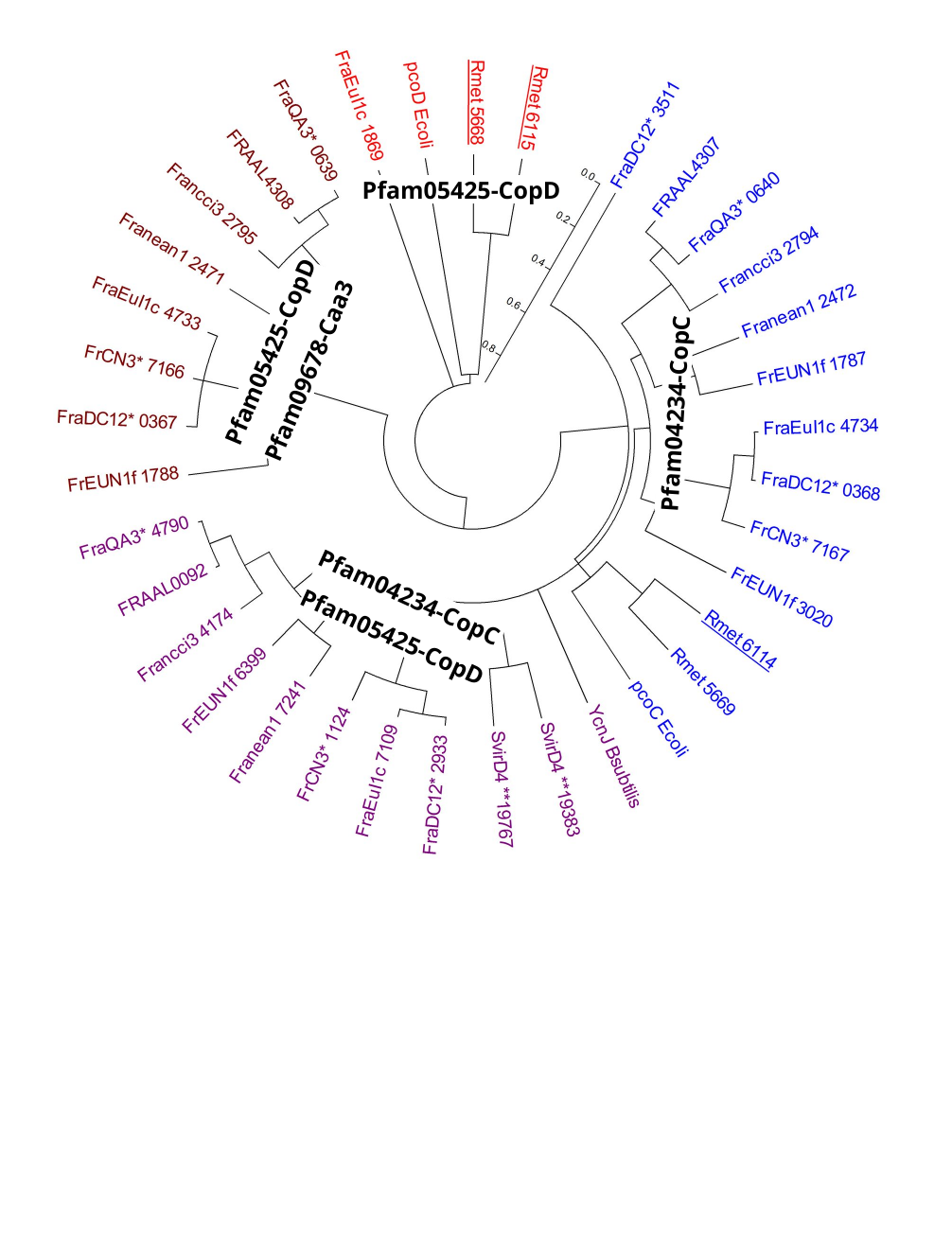

Pfam05425-CopD
Pfam05425-CopD
Pfam09678-Caa3
Pfam04234-CopC
Pfam04234-CopC
Pfam05425-CopD

Supplement: Supplementary file 6 — Additional file 6: Phylogeny of Frankia CopC and CopD proteins. Neighbor-joining tree of ClustalW aligned CopC and CopD protein sequences from Frankia and comparative organisms used in Richards et. al. 2002. Genes up-regulated with metals in compiled gene array studies are underlined. Identifying Pfam domains for each group of proteins was also included to show characteristics of Frankia CopCD proteins. * = DRAFT, ** = 0101000. (PPT 536 KB) [file 12864_2014_7073_MOESM6_ESM.ppt]

## Slide 1
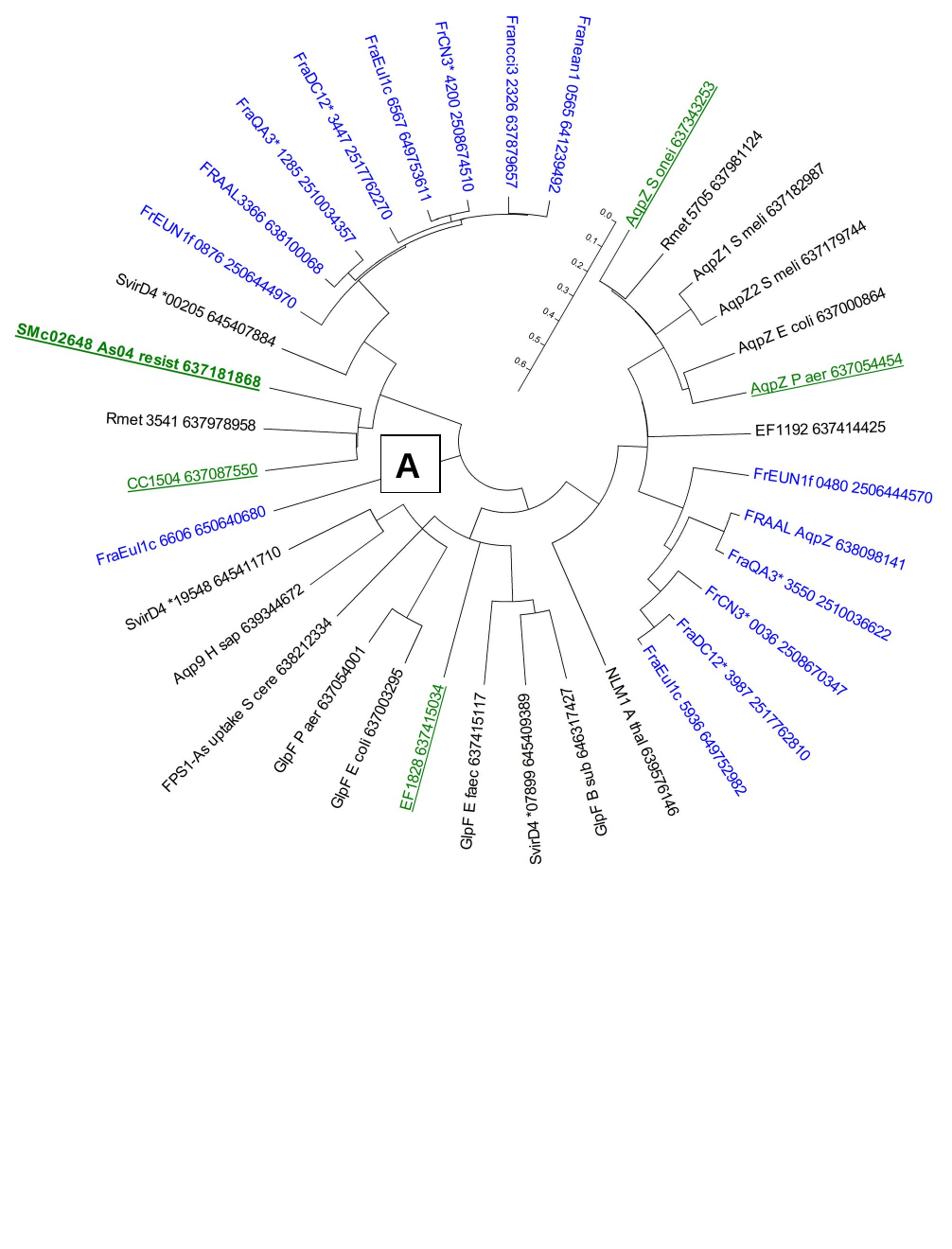

A

Supplement: Supplementary file 7 — Additional file 7: Phylogeny of Frankia arsenic transport permeases. Neighbor-joining tree of ClustalW aligned major intrinsic protein (MIP) domain (pfam00230) containing protein sequences from Frankia, from compiled gene arrays (underlined), and from several experimentally characterized genes. The novel arsenite exporting aquaporin from Sinorhizobium meliloti is in bold. Frankia sp. strain EuI1c contains a unique MIP (A) which may contribute to its sensitivity to arsenate. * = DRAFT, ** = 0101000. (PPT 791 KB) [file 12864_2014_7073_MOESM7_ESM.ppt]

## Slide 1
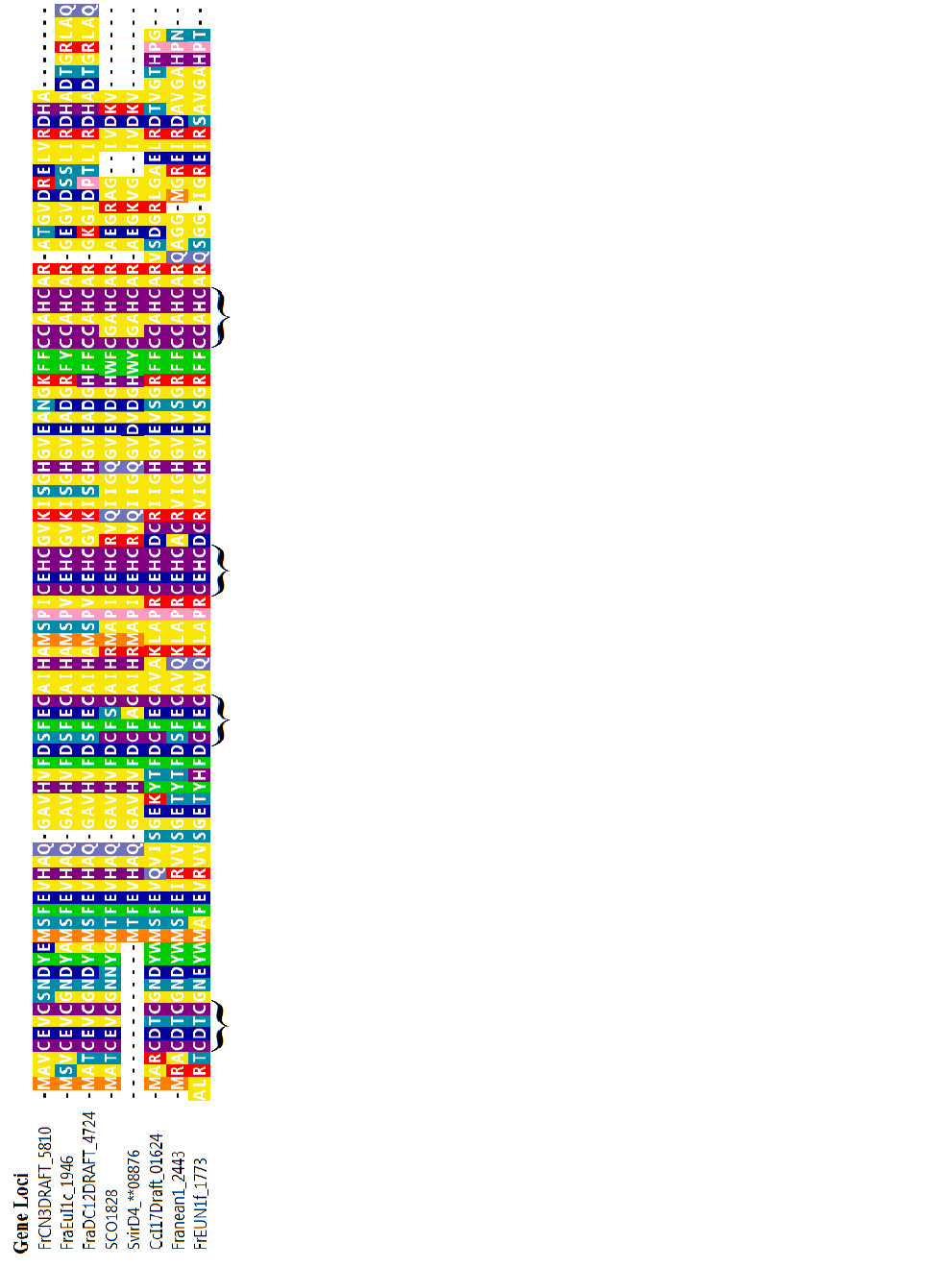

Supplement: Supplementary file 9 — Additional file 9: Protein alignment of a potential metallothionein involved in chromate resistance. ClustalW alignment of a metallothionein found only in the chromate resistant bacterial strains based Richards et. al. 2002. Brackets indicate potential metal binding motifs. Amino acids are colored by characteristic: dark blue are negatively charged (D/E), purple are uncharged metal-binding (C/H), red are positively charged (K/R), pink are cyclic (P), orange are metal-binding and hydrophobic (M), yellow are hydrophobic (L/I/V/M/G/A), green are aromatic (F/W/Y), light blue are polar (S/T/N/Q). (PPT 80 KB) [file 12864_2014_7073_MOESM9_ESM.ppt]

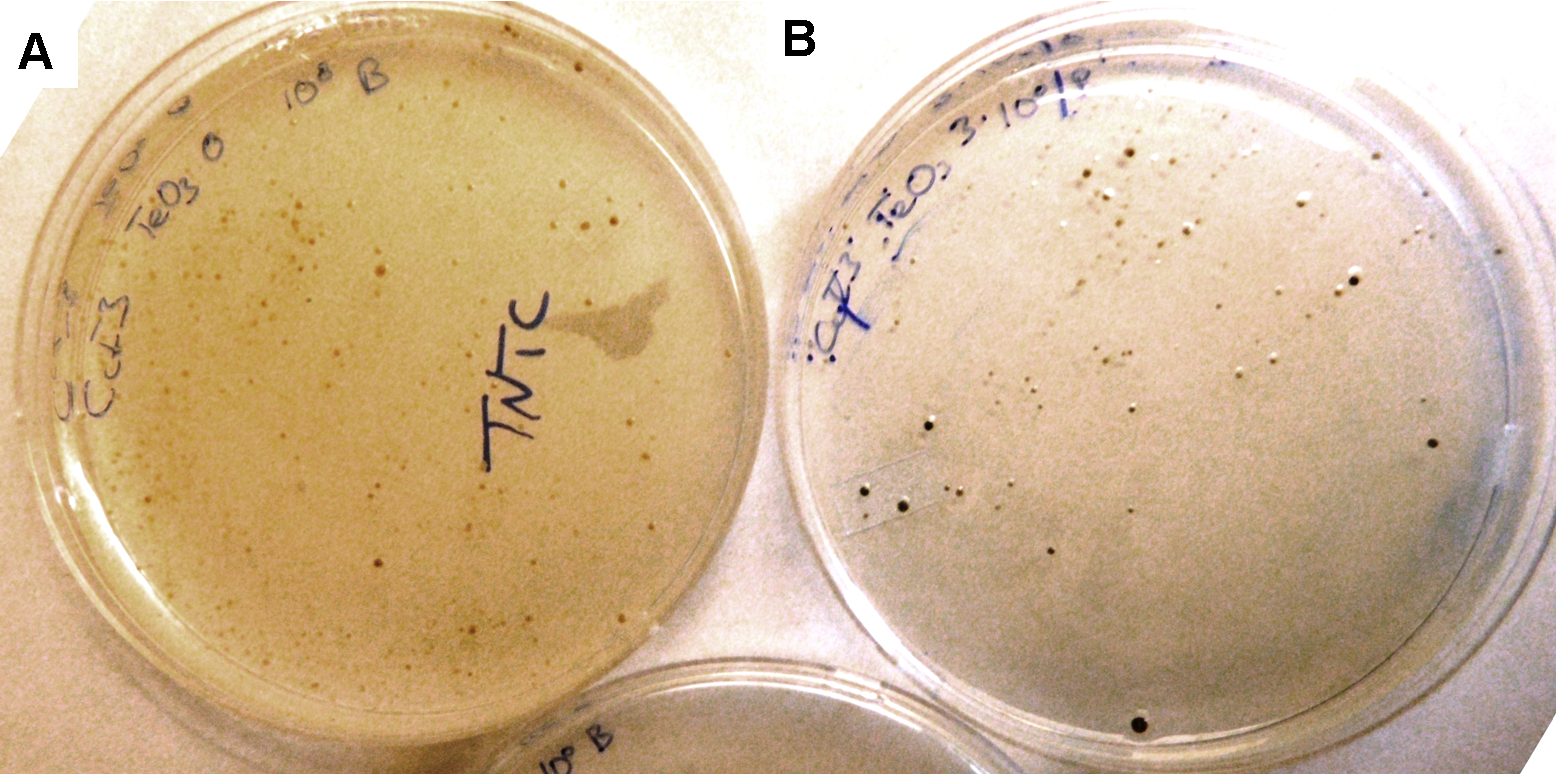

Supplement: Supplementary file 19 — Additional file 19: Confirmation of Frankia resistance and reduction of tellurite based on genome prediction. Frankia sp. strain CcI3 was grown on solid minimal media containing either [A] 0 mM or [B] 3 mM potassium tellurite. As predicted by the presence of several tellurite resistance and reduction factors in the Frankia genome, strain CcI3 was able to grow in the presence of tellurite and reduce it to elemental tellurium (black precipitate). (TIFF 4 MB) [file 12864_2014_7073_MOESM19_ESM.tiff]

## Slide 1
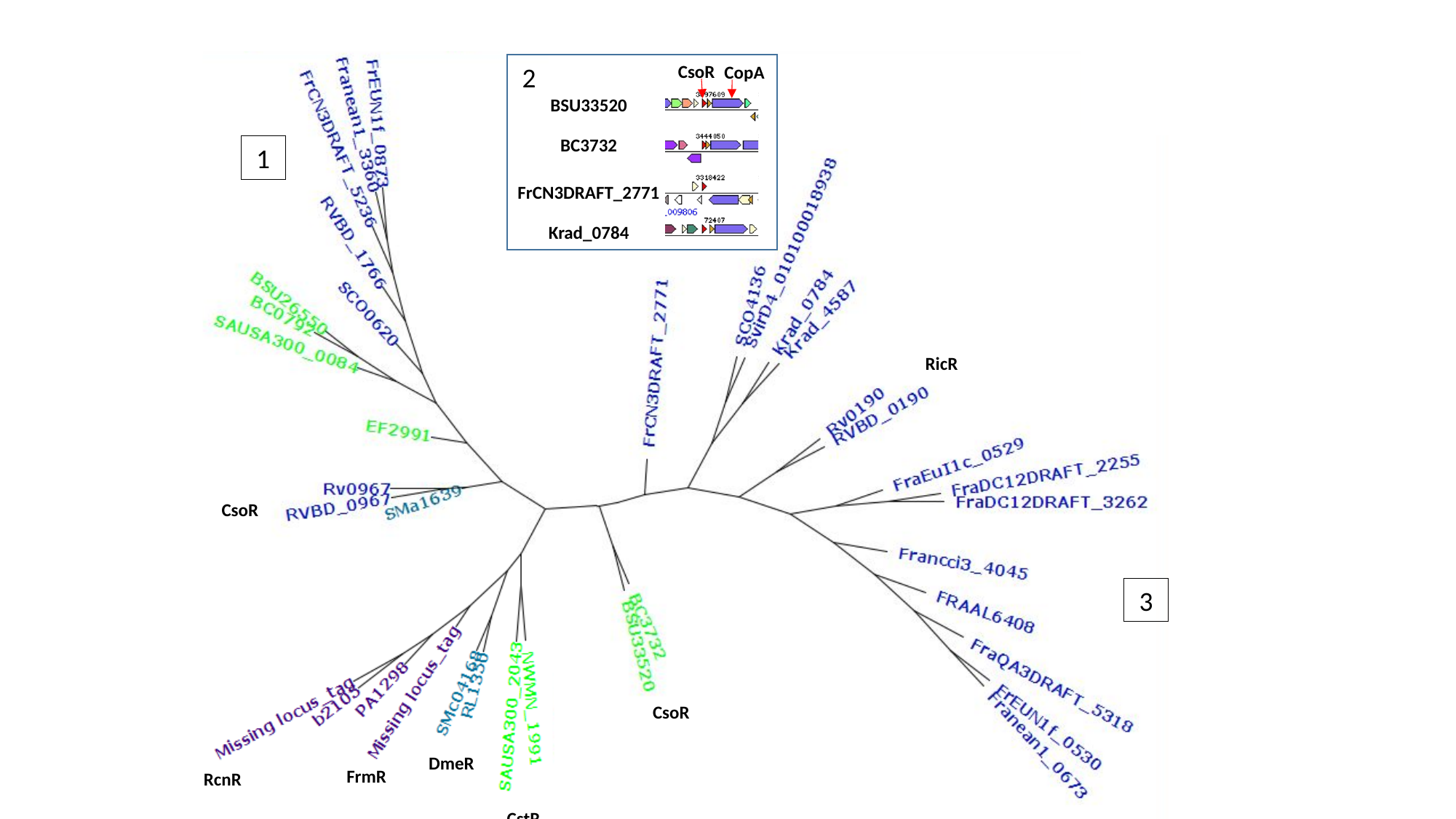

RicR
CsoR
CsoR
DmeR
FrmR
RcnR
CstR
CsoR
CopA
BSU33520
BC3732
FrCN3DRAFT_2771
Krad_0784
2
1
3

Supplement: Supplementary file 22 — Additional file 22: Phylogenetic analysis of the CsoR family proteins in Frankia. Neighbor-joining tree of Clustal Ω aligned CsoR proteins (pfam02583) containing protein sequences from Frankia, characterized members of CsoR-family, and proteins from the 18 comparative organisms (Additional file 10). 1. Uncharacterized CsoR-like proteins. 2. True CsoR proteins. The synteny between characterized CsoR (BSU33420) and the other orthologs is displayed, showing the regulatory target, copper-exporting CopA. 3. Copper regulating RicR proteins. (PPTX 689 KB) [file 12864_2014_7073_MOESM22_ESM.pptx]
