# Supplementary material for: The ins and outs of metal homeostasis by the root nodule actinobacterium Frankia
Source: BMC Genomics. 2014 Dec 12;15:1092. doi: 10.1186/1471-2164-15-1092 (PMC4531530; doi:10.1186/1471-2164-15-1092)
Supplement: Supplementary file 8 — Additional file 8: Gene neighborhood synteny of Frankia -type Cu 2+ -ATPases. The region contains genes for electron transport chain components [heme B, cytochrome C, ubiquinone (CoQ)/menaquinone (VitK)] and copper sequestration to the cell surface [phosphate metabolism and cell surface modification genes]. Association of CopA with this region suggests a dual purpose of decreasing heme disruption by cytoplasmic copper and delivering copper to the ETC complex IV. CopA in Frankia strains EAN, EUN, and CopZA in DC12 have been transposed to a different region in these genomes. (PPT 97 KB) [file 12864_2014_7073_MOESM8_ESM.ppt]

## Slide 1
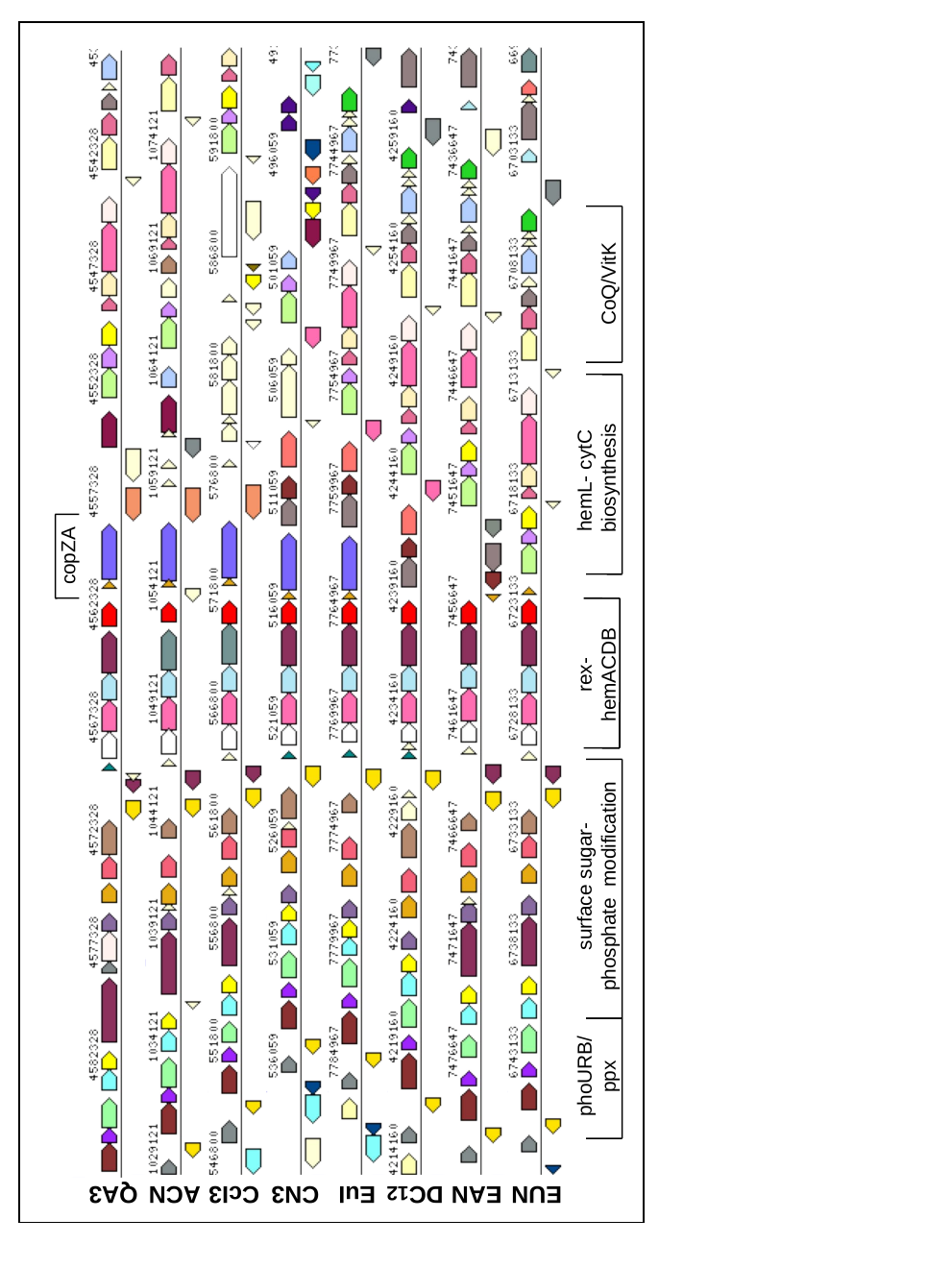

ACN
CcI3
CN3
EuI
DC12
EAN
EUN
QA3
CoQ/VitK
hemL- cytC biosynthesis
copZA
rex- hemACDB
surface sugar-phosphate modification
phoURB/ ppx
