# Supplementary material for: The ins and outs of metal homeostasis by the root nodule actinobacterium Frankia
Source: BMC Genomics. 2014 Dec 12;15:1092. doi: 10.1186/1471-2164-15-1092 (PMC4531530; doi:10.1186/1471-2164-15-1092)
Supplement: Supplementary file 11 — Additional file 11: Frankia sp. strain CcI3 metal homeostasis mechanisms. Schematic diagram of known and putative metal homeostasis systems in Frankia CcI3. Loci containing identifying domains (see Additional file 10) for metal ion uptake transporters, chaperones, modification enzymes, efflux transporters, and surface binding protein and efflux systems are shown (left to right) with arrows to indicate the flow of metals through the cell. Information at the bottom indicates whether the strain is symbiotic with host plants (Sym+/-), is a diazotroph (N2-fix+/-), and whether the strain is resistant (r) or sensitive (s) to a particular metal. (PPT 184 KB) [file 12864_2014_7073_MOESM11_ESM.ppt]

## Slide 1
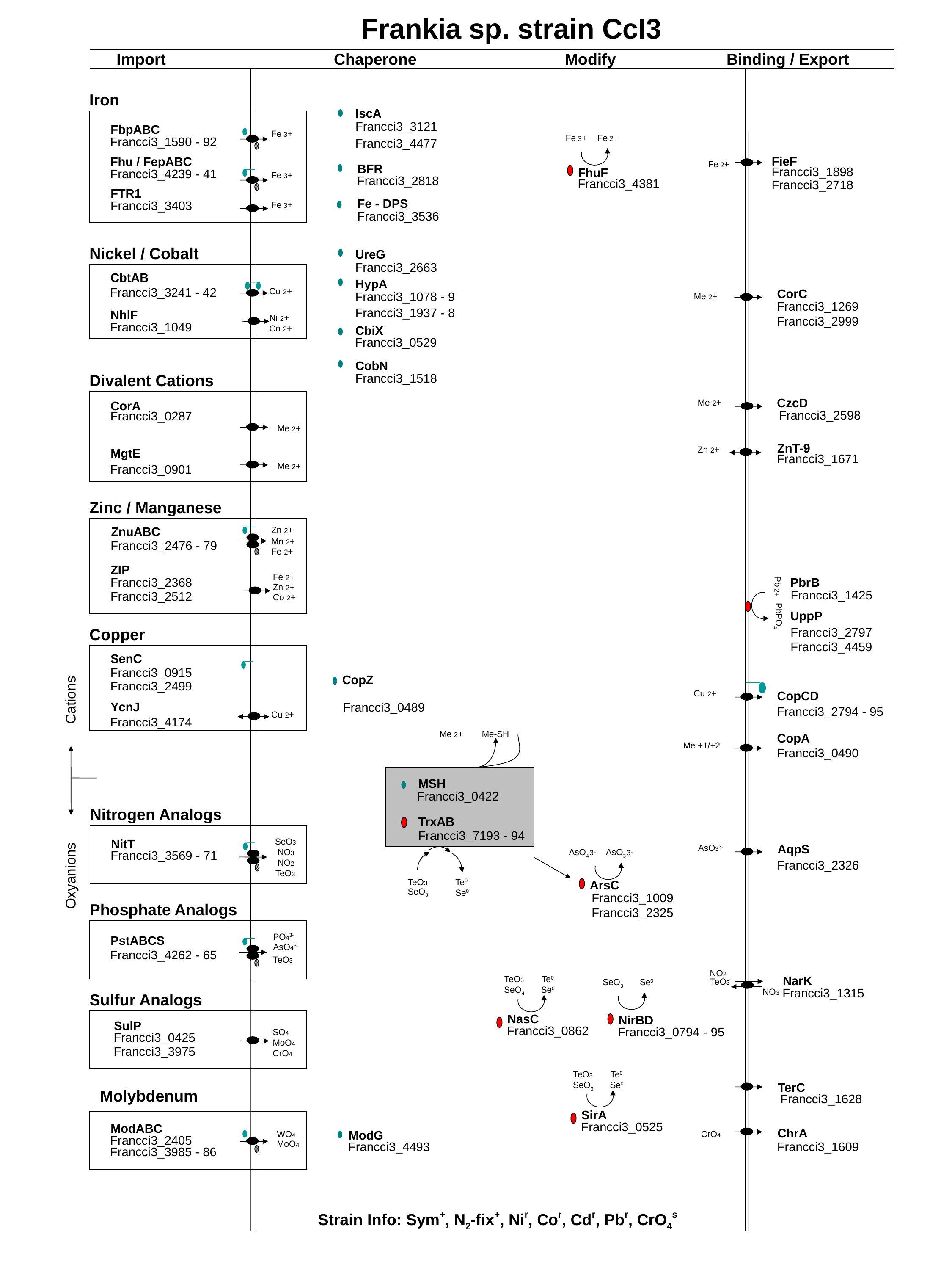

Frankia sp. strain CcI3
 Import 	 Chaperone 	 Modify 	 Binding / Export
Iron
IscA
Francci3_3121
FbpABC
Fe 3+
Francci3_4477
Fe 3+
Fe 2+
Francci3_1590 - 92
FieF
Fhu / FepABC
Fe 2+
BFR
Francci3_1898
FhuF
Francci3_4239 - 41
Fe 3+
Francci3_2818
Francci3_4381
Francci3_2718
FTR1
Fe - DPS
Francci3_3403
Fe 3+
Francci3_3536
Nickel / Cobalt
UreG
Francci3_2663
CbtAB
HypA
Francci3_3241 - 42
Co 2+
Francci3_1078 - 9
CorC
Me 2+
Francci3_1269
Francci3_1937 - 8
NhlF
Ni 2+
Francci3_2999
Francci3_1049
Co 2+
CbiX
Francci3_0529
CobN
Francci3_1518
Divalent Cations
CzcD
Me 2+
CorA
Francci3_2598
Francci3_0287
Me 2+
ZnT-9
Zn 2+
MgtE
Francci3_1671
Me 2+
Francci3_0901
Zinc / Manganese
ZnuABC
Francci3_2476 - 79
Zn 2+
Mn 2+
Fe 2+
ZIP
Fe 2+
Francci3_2368
PbrB
Pb 2+
PbPO4
Zn 2+
Francci3_1425
Francci3_2512
Co 2+
UppP
Copper
Francci3_2797
Francci3_4459
SenC
Francci3_0915
CopZ
Francci3_2499
Cations
Cu 2+
CopCD
YcnJ
Francci3_0489
Francci3_2794 - 95
Cu 2+
Francci3_4174
Me 2+
Me-SH
CopA
Me +1/+2
Francci3_0490
MSH
Francci3_0422
Nitrogen Analogs
TrxAB
Francci3_7193 - 94
NitT
SeO3
AqpS
AsO33-
NO3
TeO3
Te0
SeO3
Se0
AsO4 3-
AsO3 3-
Francci3_3569 - 71
NO2
Francci3_2326
Oxyanions
TeO3
ArsC
Francci3_1009
Phosphate Analogs
Francci3_2325
PO43-
PstABCS
AsO43-
Francci3_4262 - 65
TeO3
NO2
TeO3
NarK
TeO3
Te0
SeO3
Se0
NO3
SeO4
Se0
Francci3_1315
Sulfur Analogs
NasC
NirBD
SulP
Francci3_0862
Francci3_0794 - 95
SO4
Francci3_0425
MoO4
Francci3_3975
CrO4
TeO3
Te0
SeO3
Se0
TerC
Molybdenum
Francci3_1628
SirA
Francci3_0525
ModABC
ChrA
ModG
WO4
CrO4
Francci3_2405
MoO4
Francci3_4493
Francci3_1609
Francci3_3985 - 86
Strain Info: Sym+, N2-fix+, Nir, Cor, Cdr, Pbr, CrO4s
