# Supplementary material for: The ins and outs of metal homeostasis by the root nodule actinobacterium Frankia
Source: BMC Genomics. 2014 Dec 12;15:1092. doi: 10.1186/1471-2164-15-1092 (PMC4531530; doi:10.1186/1471-2164-15-1092)
Supplement: Supplementary file 12 — Additional file 12: Frankia sp. strain QA3 metal homeostasis mechanisms. Schematic diagram of known and putative metal homeostasis systems in Frankia sp. strain QA3. Loci containing identifying domains (see Additional file 10) for metal ion uptake transporters, chaperones, modification enzymes, efflux transporters, and surface binding protein and efflux systems are shown (left to right) with arrows to indicate the flow of metals through the cell. Information at the bottom indicates whether the strain is symbiotic with host plants (Sym+/-), is a diazotroph (N2-fix+/-), and whether the strain is resistant (r) or sensitive (s) to a particular metal. * = DRAFT. (PPT 184 KB) [file 12864_2014_7073_MOESM12_ESM.ppt]

## Slide 1
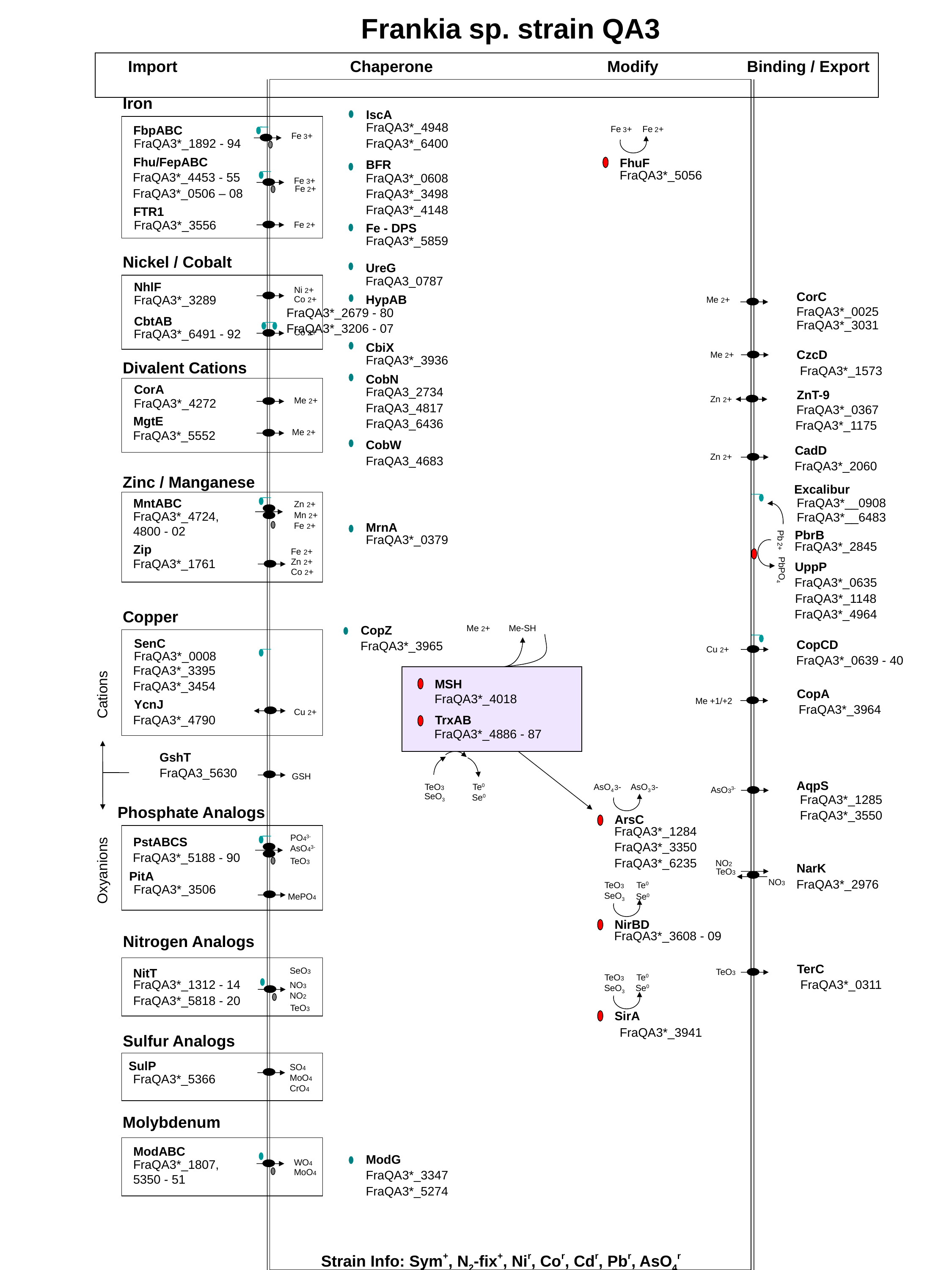

Frankia sp. strain QA3
 Import Chaperone 	 Modify Binding / Export
Iron
IscA
FraQA3*_4948
Fe 3+
Fe 2+
FbpABC
Fe 3+
FraQA3*_6400
FraQA3*_1892 - 94
FhuF
Fhu/FepABC
BFR
FraQA3*_5056
FraQA3*_4453 - 55
FraQA3*_0608
Fe 3+
Fe 2+
FraQA3*_0506 – 08
FraQA3*_3498
FraQA3*_4148
FTR1
FraQA3*_3556
Fe 2+
Fe - DPS
FraQA3*_5859
Nickel / Cobalt
UreG
FraQA3_0787
NhlF
Ni 2+
CorC
HypAB
FraQA3*_3289
Co 2+
Me 2+
FraQA3*_0025
FraQA3*_2679 - 80
CbtAB
FraQA3*_3031
FraQA3*_3206 - 07
FraQA3*_6491 - 92
Co 2+
CbiX
CzcD
Me 2+
FraQA3*_3936
Divalent Cations
FraQA3*_1573
CobN
CorA
FraQA3_2734
ZnT-9
Zn 2+
Me 2+
FraQA3*_4272
FraQA3_4817
FraQA3*_0367
MgtE
FraQA3_6436
FraQA3*_1175
Me 2+
FraQA3*_5552
CobW
CadD
Zn 2+
FraQA3_4683
FraQA3*_2060
Zinc / Manganese
Excalibur
MntABC
FraQA3*__0908
Zn 2+
FraQA3*_4724, 4800 - 02
Mn 2+
FraQA3*__6483
MrnA
Fe 2+
PbrB
Pb 2+
PbPO4
FraQA3*_0379
FraQA3*_2845
Zip
Fe 2+
FraQA3*_1761
UppP
Zn 2+
Co 2+
FraQA3*_0635
FraQA3*_1148
Copper
FraQA3*_4964
CopZ
Me 2+
Me-SH
SenC
FraQA3*_3965
CopCD
Cu 2+
FraQA3*_0008
FraQA3*_0639 - 40
FraQA3*_3395
MSH
Cations
FraQA3*_3454
CopA
FraQA3*_4018
Me +1/+2
YcnJ
FraQA3*_3964
Cu 2+
FraQA3*_4790
TrxAB
FraQA3*_4886 - 87
GshT
TeO3
Te0
SeO3
Se0
FraQA3_5630
GSH
AqpS
AsO4 3-
AsO3 3-
AsO33-
FraQA3*_1285
Phosphate Analogs
FraQA3*_3550
ArsC
FraQA3*_1284
PO43-
PstABCS
FraQA3*_3350
AsO43-
FraQA3*_5188 - 90
TeO3
FraQA3*_6235
NO2
NarK
Oxyanions
TeO3
PitA
NO3
FraQA3*_2976
TeO3
Te0
FraQA3*_3506
SeO3
MePO4
Se0
NirBD
FraQA3*_3608 - 09
Nitrogen Analogs
TerC
NitT
SeO3
TeO3
TeO3
Te0
FraQA3*_1312 - 14
FraQA3*_0311
NO3
SeO3
Se0
NO2
FraQA3*_5818 - 20
TeO3
SirA
FraQA3*_3941
Sulfur Analogs
SulP
SO4
FraQA3*_5366
MoO4
CrO4
Molybdenum
ModABC
ModG
FraQA3*_1807, 5350 - 51
WO4
MoO4
FraQA3*_3347
FraQA3*_5274
Strain Info: Sym+, N2-fix+, Nir, Cor, Cdr, Pbr, AsO4r
