# Supplementary material for: The ins and outs of metal homeostasis by the root nodule actinobacterium Frankia
Source: BMC Genomics. 2014 Dec 12;15:1092. doi: 10.1186/1471-2164-15-1092 (PMC4531530; doi:10.1186/1471-2164-15-1092)
Supplement: Supplementary file 13 — Additional file 13: Frankia alni ACN14a metal homeostasis mechanisms. Schematic diagram of known and putative metal homeostasis systems in Frankia alni ACN14a. Loci containing identifying domains (see Additional file 10) for metal ion uptake transporters, chaperones, modification enzymes, efflux transporters, and surface binding protein and efflux systems are shown (left to right) with arrows to indicate the flow of metals through the cell. Information at the bottom indicates whether the strain is symbiotic with host plants (Sym+/-), is a diazotroph (N2-fix+/-), and whether the strain is resistant (r) or sensitive (s) to a particular metal. (PPT 183 KB) [file 12864_2014_7073_MOESM13_ESM.ppt]

## Slide 1
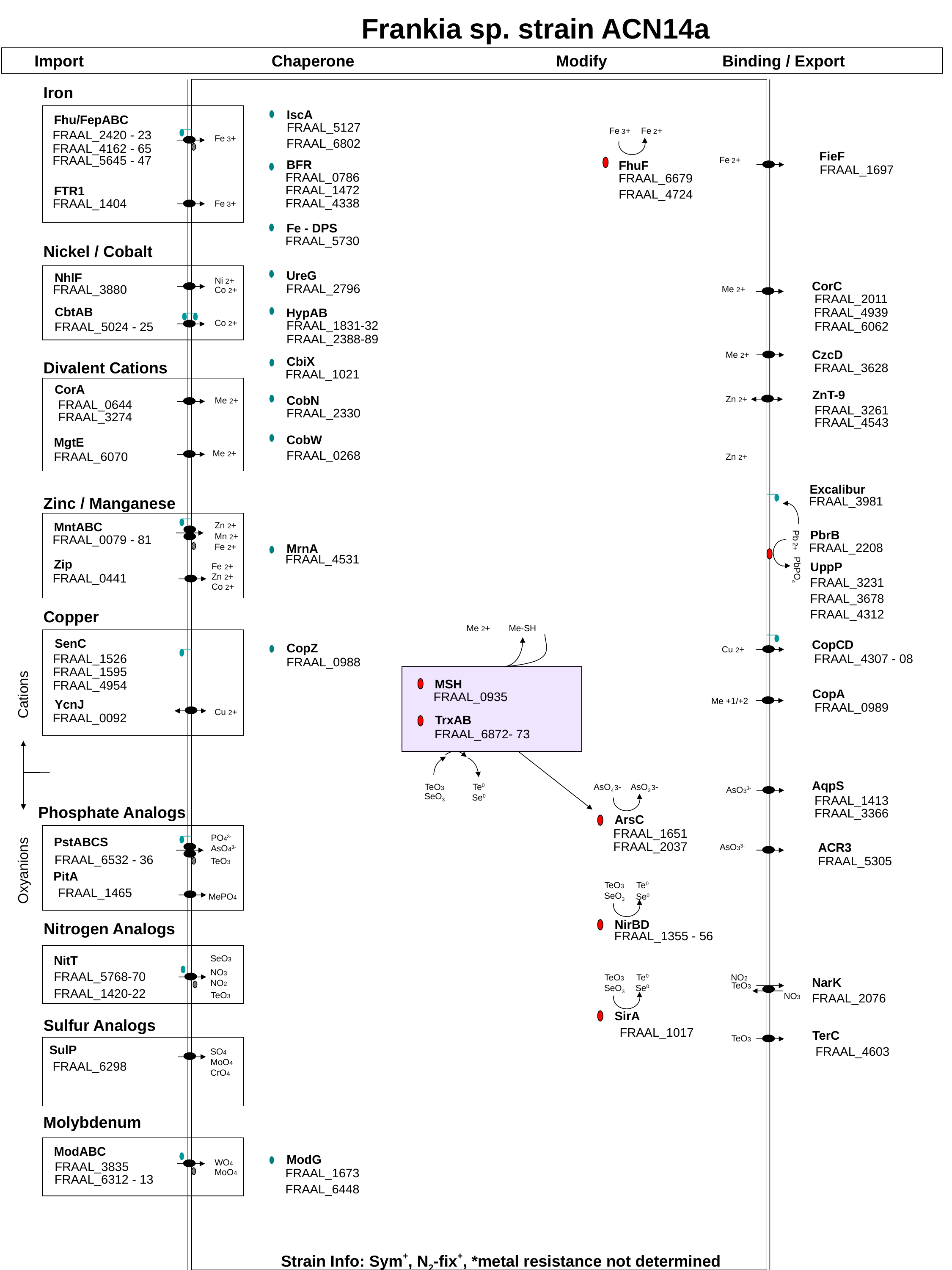

Frankia sp. strain ACN14a
 Import	 Chaperone 		 Modify 	 Binding / Export
Iron
IscA
Fhu/FepABC
FRAAL_5127
Fe 3+
Fe 2+
FRAAL_2420 - 23
Fe 3+
FRAAL_6802
FRAAL_4162 - 65
FieF
FRAAL_5645 - 47
BFR
Fe 2+
FhuF
FRAAL_6679
FRAAL_1697
FRAAL_0786
FTR1
FRAAL_1472
FRAAL_4724
Fe 3+
FRAAL_4338
FRAAL_1404
Fe - DPS
FRAAL_5730
Nickel / Cobalt
UreG
NhlF
Ni 2+
CorC
FRAAL_2796
Me 2+
Co 2+
FRAAL_3880
FRAAL_2011
HypAB
CbtAB
FRAAL_4939
Co 2+
FRAAL_1831-32
FRAAL_6062
FRAAL_5024 - 25
FRAAL_2388-89
CzcD
Me 2+
CbiX
Divalent Cations
FRAAL_3628
FRAAL_1021
CorA
ZnT-9
CobN
Zn 2+
Me 2+
FRAAL_0644
FRAAL_2330
FRAAL_3261
FRAAL_3274
FRAAL_4543
CobW
MgtE
Me 2+
FRAAL_0268
FRAAL_6070
Zn 2+
Excalibur
Zinc / Manganese
FRAAL_3981
Zn 2+
MntABC
PbrB
Pb 2+
PbPO4
Mn 2+
FRAAL_0079 - 81
MrnA
Fe 2+
FRAAL_2208
FRAAL_4531
Zip
UppP
Fe 2+
FRAAL_0441
Zn 2+
FRAAL_3231
Co 2+
FRAAL_3678
Copper
FRAAL_4312
Me 2+
Me-SH
SenC
CopCD
CopZ
Cu 2+
FRAAL_1526
FRAAL_0988
FRAAL_4307 - 08
FRAAL_1595
MSH
Cations
FRAAL_4954
CopA
FRAAL_0935
Me +1/+2
YcnJ
FRAAL_0989
Cu 2+
TrxAB
FRAAL_0092
FRAAL_6872- 73
TeO3
Te0
SeO3
Se0
AqpS
AsO4 3-
AsO3 3-
AsO33-
FRAAL_1413
Phosphate Analogs
FRAAL_3366
ArsC
FRAAL_1651
PO43-
PstABCS
FRAAL_2037
AsO43-
ACR3
AsO33-
FRAAL_6532 - 36
TeO3
FRAAL_5305
Oxyanions
PitA
TeO3
Te0
FRAAL_1465
SeO3
MePO4
Se0
NirBD
Nitrogen Analogs
FRAAL_1355 - 56
NitT
SeO3
NO3
FRAAL_5768-70
TeO3
Te0
NO2
NarK
NO2
TeO3
SeO3
Se0
FRAAL_1420-22
TeO3
NO3
FRAAL_2076
SirA
Sulfur Analogs
FRAAL_1017
TerC
TeO3
FRAAL_4603
SulP
SO4
MoO4
FRAAL_6298
CrO4
Molybdenum
ModABC
ModG
WO4
FRAAL_3835
MoO4
FRAAL_1673
FRAAL_6312 - 13
FRAAL_6448
Strain Info: Sym+, N2-fix+, *metal resistance not determined
