# Supplementary material for: The ins and outs of metal homeostasis by the root nodule actinobacterium Frankia
Source: BMC Genomics. 2014 Dec 12;15:1092. doi: 10.1186/1471-2164-15-1092 (PMC4531530; doi:10.1186/1471-2164-15-1092)
Supplement: Supplementary file 14 — Additional file 14: Frankia sp. strain EUN1f metal homeostasis mechanisms. Schematic diagram of known and putative metal homeostasis systems in Frankia sp. strain EUN. Loci containing identifying domains (see Additional file 10) for metal ion uptake transporters, chaperones, modification enzymes, efflux transporters, and surface binding protein and efflux systems are shown (left to right) with arrows to indicate the flow of metals through the cell. Information at the bottom indicates whether the strain is symbiotic with host plants (Sym+/-), is a diazotroph (N2-fix+/-), and whether the strain is resistant (r) or sensitive (s) to a particular metal. (PPT 185 KB) [file 12864_2014_7073_MOESM14_ESM.ppt]

## Slide 1
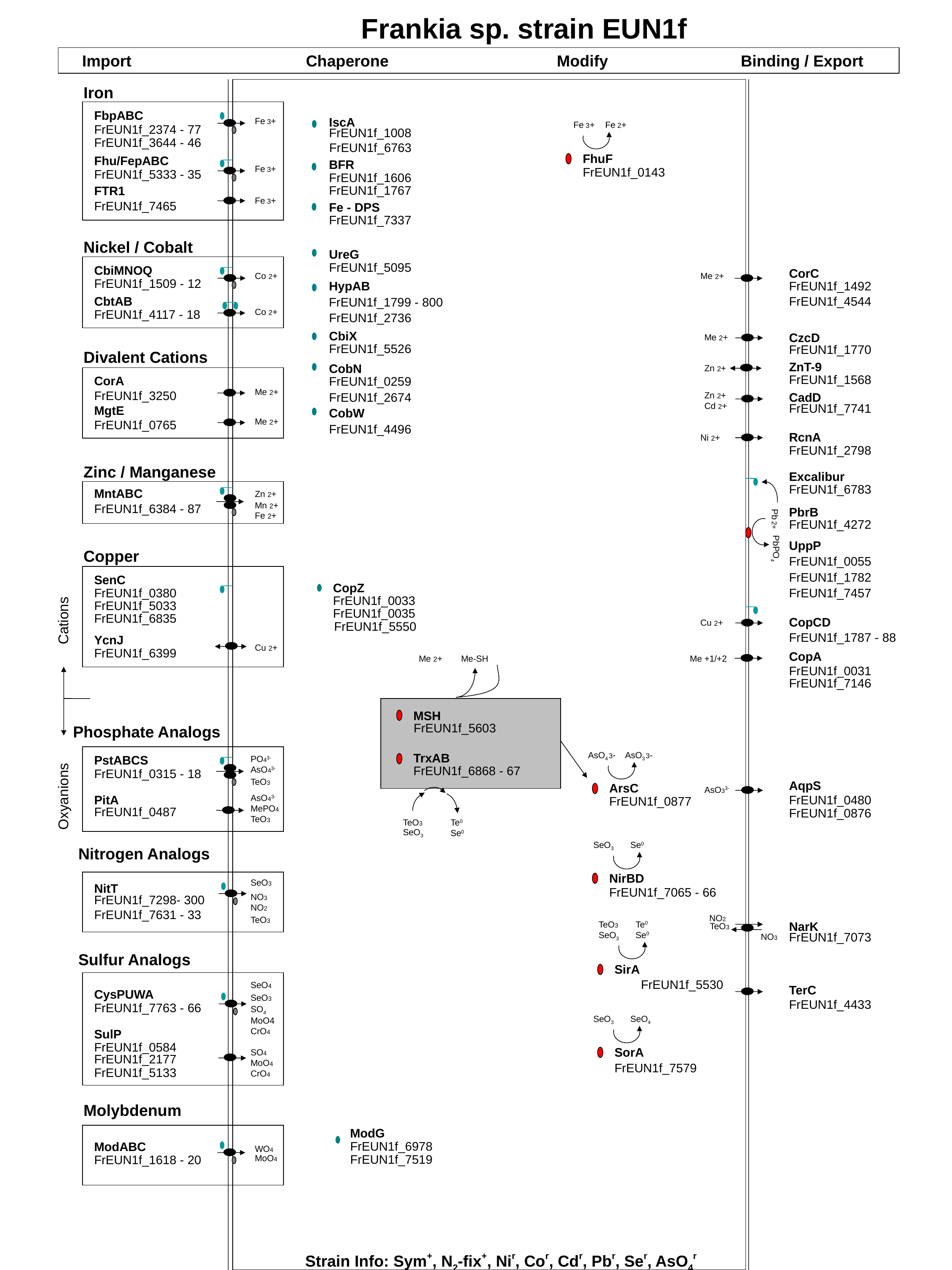

Frankia sp. strain EUN1f
 Import	 Chaperone Modify Binding / Export
Iron
FbpABC
IscA
Fe 3+
Fe 3+
Fe 2+
FrEUN1f_2374 - 77
FrEUN1f_1008
FrEUN1f_3644 - 46
FrEUN1f_6763
FhuF
Fhu/FepABC
BFR
Fe 3+
FrEUN1f_0143
FrEUN1f_5333 - 35
FrEUN1f_1606
FrEUN1f_1767
FTR1
Fe 3+
FrEUN1f_7465
Fe - DPS
FrEUN1f_7337
Nickel / Cobalt
UreG
FrEUN1f_5095
CbiMNOQ
CorC
Me 2+
Co 2+
FrEUN1f_1509 - 12
HypAB
FrEUN1f_1492
FrEUN1f_4544
FrEUN1f_1799 - 800
CbtAB
Co 2+
FrEUN1f_4117 - 18
FrEUN1f_2736
CbiX
CzcD
Me 2+
FrEUN1f_5526
FrEUN1f_1770
Divalent Cations
ZnT-9
CobN
Zn 2+
FrEUN1f_1568
FrEUN1f_0259
CorA
Me 2+
FrEUN1f_3250
FrEUN1f_2674
Zn 2+
CadD
Cd 2+
FrEUN1f_7741
CobW
MgtE
Me 2+
FrEUN1f_0765
FrEUN1f_4496
Ni 2+
RcnA
FrEUN1f_2798
Zinc / Manganese
Excalibur
FrEUN1f_6783
MntABC
Zn 2+
Mn 2+
FrEUN1f_6384 - 87
PbrB
Pb 2+
PbPO4
Fe 2+
FrEUN1f_4272
UppP
Copper
FrEUN1f_0055
FrEUN1f_1782
SenC
CopZ
FrEUN1f_0380
FrEUN1f_7457
FrEUN1f_0033
FrEUN1f_5033
Cations
FrEUN1f_0035
FrEUN1f_6835
Cu 2+
CopCD
FrEUN1f_5550
FrEUN1f_1787 - 88
YcnJ
Cu 2+
FrEUN1f_6399
CopA
Me 2+
Me-SH
Me +1/+2
FrEUN1f_0031
FrEUN1f_7146
MSH
FrEUN1f_5603
Phosphate Analogs
AsO4 3-
AsO3 3-
TrxAB
PO43-
PstABCS
FrEUN1f_6868 - 67
AsO43-
FrEUN1f_0315 - 18
TeO3
AqpS
ArsC
AsO33-
Oxyanions
TeO3
Te0
SeO3
Se0
FrEUN1f_0480
PitA
AsO43-
FrEUN1f_0877
MePO4
FrEUN1f_0487
FrEUN1f_0876
TeO3
SeO3
Se0
Nitrogen Analogs
NirBD
SeO3
NitT
FrEUN1f_7065 - 66
NO3
FrEUN1f_7298- 300
NO2
FrEUN1f_7631 - 33
TeO3
NO2
TeO3
Te0
NarK
TeO3
SeO3
Se0
FrEUN1f_7073
NO3
Sulfur Analogs
SirA
FrEUN1f_5530
SeO4
TerC
CysPUWA
SeO3
FrEUN1f_4433
FrEUN1f_7763 - 66
SO4
SeO3
SeO4
MoO4
CrO4
SulP
FrEUN1f_0584
SorA
SO4
FrEUN1f_2177
MoO4
FrEUN1f_7579
FrEUN1f_5133
CrO4
Molybdenum
ModG
FrEUN1f_6978
ModABC
WO4
FrEUN1f_7519
FrEUN1f_1618 - 20
MoO4
Strain Info: Sym+, N2-fix+, Nir, Cor, Cdr, Pbr, Ser, AsO4r
