# Supplementary material for: The ins and outs of metal homeostasis by the root nodule actinobacterium Frankia
Source: BMC Genomics. 2014 Dec 12;15:1092. doi: 10.1186/1471-2164-15-1092 (PMC4531530; doi:10.1186/1471-2164-15-1092)
Supplement: Supplementary file 15 — Additional file 15: Frankia sp. strain EAN1pec metal homeostasis mechanisms. Schematic diagram of known and putative metal homeostasis systems in Frankia sp. strain EAN1pec. Loci containing identifying domains (see Additional file 10) for metal ion uptake transporters, chaperones, modification enzymes, efflux transporters, and surface binding protein and efflux systems are shown (left to right) with arrows to indicate the flow of metals through the cell. Information at the bottom indicates whether the strain is symbiotic with host plants (Sym+/-), is a diazotroph (N2-fix+/-), and whether the strain is resistant (r) or sensitive (s) to a particular metal. (PPT 192 KB) [file 12864_2014_7073_MOESM15_ESM.ppt]

## Slide 1
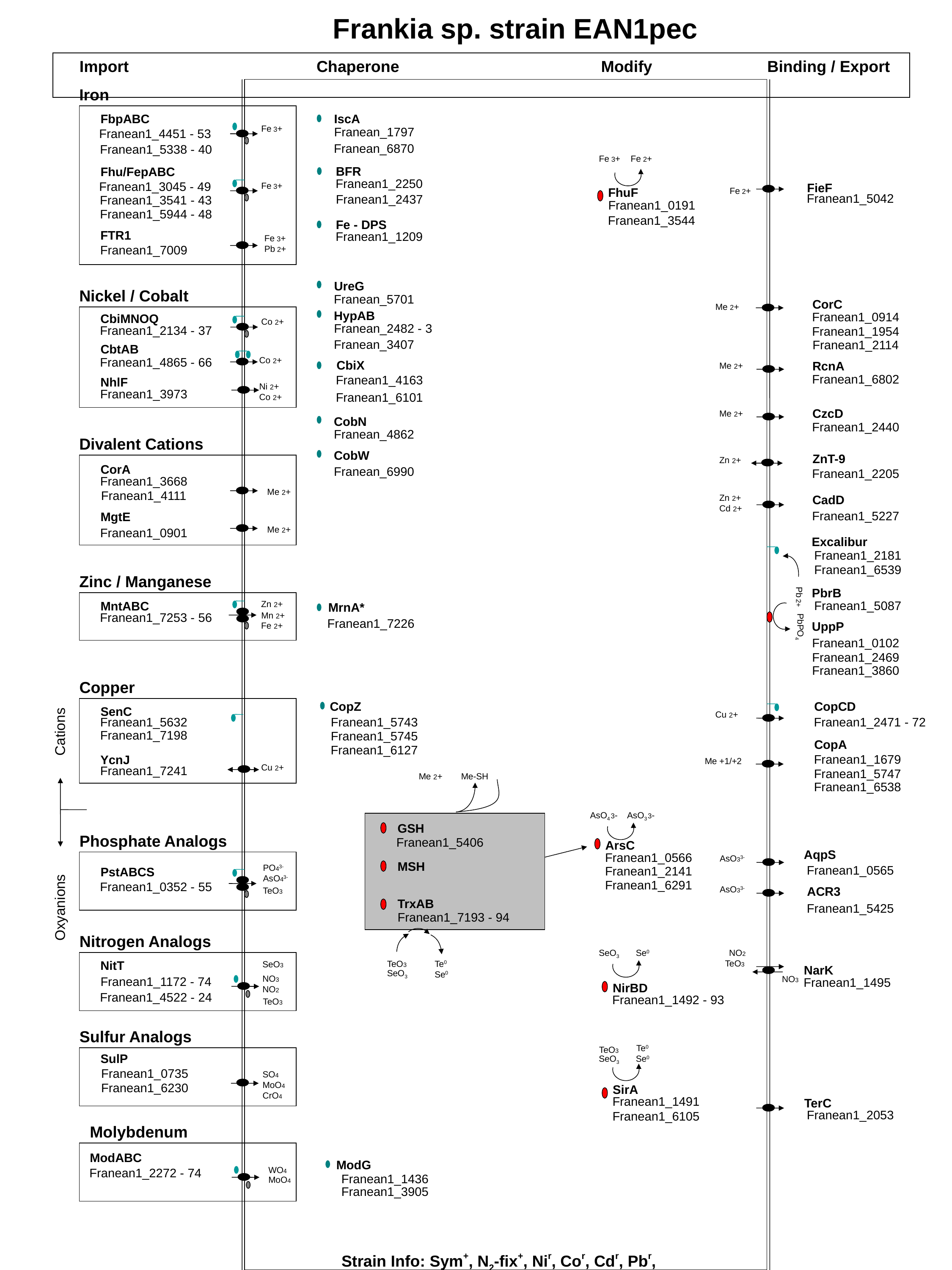

Frankia sp. strain EAN1pec
 Import	 Chaperone 		 Modify 	 Binding / Export
Iron
IscA
FbpABC
Franean_1797
Fe 3+
Franean1_4451 - 53
Franean_6870
Franean1_5338 - 40
Fe 3+
Fe 2+
BFR
Fhu/FepABC
Franean1_2250
Franean1_3045 - 49
FieF
Fe 3+
FhuF
Fe 2+
Franean1_5042
Franean1_2437
Franean1_3541 - 43
Franean1_0191
Franean1_5944 - 48
Franean1_3544
Fe - DPS
FTR1
Franean1_1209
Fe 3+
Franean1_7009
Pb 2+
UreG
Nickel / Cobalt
Franean_5701
CorC
Me 2+
HypAB
Franean1_0914
CbiMNOQ
Co 2+
Franean_2482 - 3
Franean1_2134 - 37
Franean1_1954
Franean_3407
Franean1_2114
CbtAB
Franean1_4865 - 66
Co 2+
CbiX
RcnA
Me 2+
Franean1_6802
Franean1_4163
NhlF
Ni 2+
Franean1_3973
Franean1_6101
Co 2+
CzcD
Me 2+
CobN
Franean1_2440
Franean_4862
Divalent Cations
CobW
ZnT-9
Zn 2+
Franean_6990
CorA
Franean1_2205
Franean1_3668
Me 2+
Franean1_4111
Zn 2+
CadD
Cd 2+
Franean1_5227
MgtE
Me 2+
Franean1_0901
Excalibur
Franean1_2181
Franean1_6539
Zinc / Manganese
PbrB
Pb 2+
PbPO4
Franean1_5087
MntABC
Zn 2+
MrnA*
Franean1_7253 - 56
Mn 2+
Franean1_7226
UppP
Fe 2+
Franean1_0102
Franean1_2469
Franean1_3860
Copper
CopZ
CopCD
SenC
Cu 2+
Cations
Franean1_5632
Franean1_2471 - 72
Franean1_5743
Franean1_7198
Franean1_5745
CopA
Franean1_6127
Franean1_1679
YcnJ
Me +1/+2
Cu 2+
Franean1_7241
Franean1_5747
Me 2+
Me-SH
Franean1_6538
AsO4 3-
AsO3 3-
GSH
Phosphate Analogs
Franean1_5406
ArsC
AqpS
Franean1_0566
AsO33-
MSH
PO43-
Franean1_0565
Franean1_2141
PstABCS
Franean1_6177
AsO43-
Franean1_6291
Franean1_0352 - 55
AsO33-
ACR3
TeO3
TrxAB
Oxyanions
Franean1_5425
Franean1_7193 - 94
TeO3
Te0
SeO3
Se0
Nitrogen Analogs
SeO3
Se0
NO2
NitT
TeO3
SeO3
NarK
NO3
Franean1_1172 - 74
NO3
Franean1_1495
NirBD
NO2
Franean1_4522 - 24
Franean1_1492 - 93
TeO3
Sulfur Analogs
Te0
TeO3
SulP
SeO3
Se0
Franean1_0735
SO4
MoO4
Franean1_6230
SirA
CrO4
Franean1_1491
TerC
Franean1_2053
Franean1_6105
Molybdenum
ModABC
ModG
WO4
Franean1_2272 - 74
Franean1_1436
MoO4
Franean1_3905
Strain Info: Sym+, N2-fix+, Nir, Cor, Cdr, Pbr,
