# Supplementary material for: The ins and outs of metal homeostasis by the root nodule actinobacterium Frankia
Source: BMC Genomics. 2014 Dec 12;15:1092. doi: 10.1186/1471-2164-15-1092 (PMC4531530; doi:10.1186/1471-2164-15-1092)
Supplement: Supplementary file 16 — Additional file 16: Frankia sp. strain EuI1c metal homeostasis mechanisms. Schematic diagram of known and putative metal homeostasis systems in Frankia sp. strain EuI1c. Loci containing identifying domains (see Additional file 10) for metal ion uptake transporters, chaperones, modification enzymes, efflux transporters, and surface binding protein and efflux systems are shown (left to right) with arrows to indicate the flow of metals through the cell. Information at the bottom indicates whether the strain is symbiotic with host plants (Sym+/-), is a diazotroph (N2-fix+/-), and whether the strain is resistant (r) or sensitive (s) to a particular metal. (PPT 185 KB) [file 12864_2014_7073_MOESM16_ESM.ppt]

## Slide 1
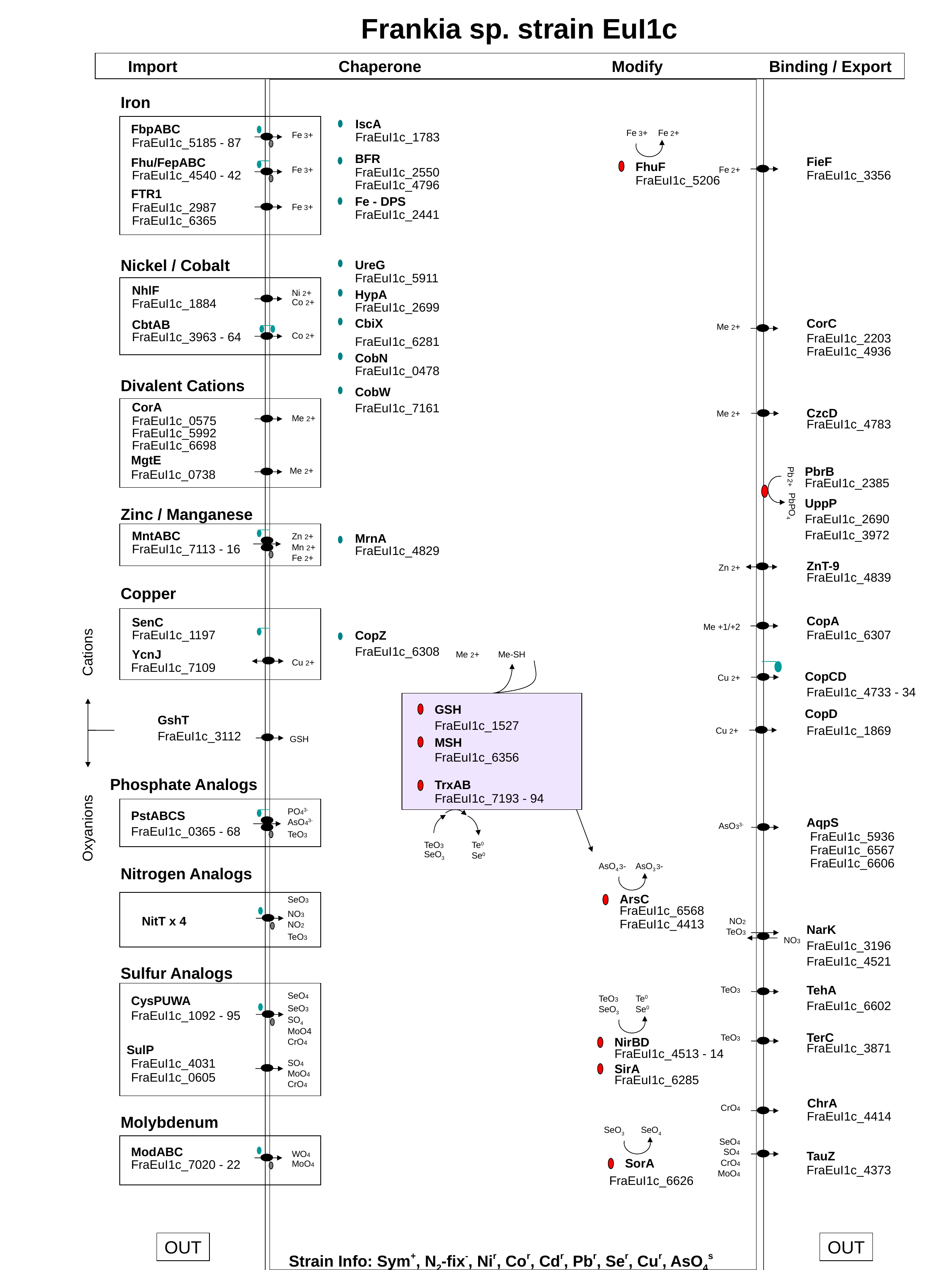

Frankia sp. strain EuI1c
 Import	 Chaperone 	 Modify Binding / Export
Iron
IscA
FbpABC
Fe 3+
Fe 2+
Fe 3+
FraEuI1c_1783
FraEuI1c_5185 - 87
BFR
FieF
Fhu/FepABC
FhuF
Fe 3+
FraEuI1c_2550
FraEuI1c_4540 - 42
Fe 2+
FraEuI1c_3356
FraEuI1c_5206
FraEuI1c_4796
FTR1
Fe - DPS
FraEuI1c_2987
Fe 3+
FraEuI1c_2441
FraEuI1c_6365
Nickel / Cobalt
UreG
FraEuI1c_5911
NhlF
HypA
Ni 2+
FraEuI1c_1884
Co 2+
FraEuI1c_2699
CbiX
CorC
CbtAB
Me 2+
FraEuI1c_3963 - 64
Co 2+
FraEuI1c_2203
FraEuI1c_6281
FraEuI1c_4936
CobN
FraEuI1c_0478
Divalent Cations
CobW
FraEuI1c_7161
CorA
CzcD
Me 2+
Me 2+
FraEuI1c_0575
FraEuI1c_4783
FraEuI1c_5992
FraEuI1c_6698
MgtE
Pb 2+
PbPO4
PbrB
Me 2+
FraEuI1c_0738
FraEuI1c_2385
UppP
Zinc / Manganese
FraEuI1c_2690
FraEuI1c_3972
MrnA
MntABC
Zn 2+
FraEuI1c_7113 - 16
Mn 2+
FraEuI1c_4829
Fe 2+
ZnT-9
Zn 2+
FraEuI1c_4839
Copper
CopA
SenC
Me +1/+2
FraEuI1c_6307
FraEuI1c_1197
CopZ
Cations
FraEuI1c_6308
YcnJ
Me 2+
Me-SH
Cu 2+
FraEuI1c_7109
CopCD
Cu 2+
FraEuI1c_4733 - 34
GSH
CopD
GshT
FraEuI1c_1527
FraEuI1c_1869
FraEuI1c_3112
Cu 2+
GSH
MSH
FraEuI1c_6356
Phosphate Analogs
TrxAB
FraEuI1c_7193 - 94
PO43-
PstABCS
TeO3
Te0
SeO3
Se0
AqpS
AsO43-
Oxyanions
FraEuI1c_0365 - 68
AsO33-
TeO3
FraEuI1c_5936
FraEuI1c_6567
FraEuI1c_6606
AsO4 3-
AsO3 3-
Nitrogen Analogs
ArsC
SeO3
FraEuI1c_6568
NO3
NitT x 4
FraEuI1c_4413
NO2
NO2
NarK
TeO3
TeO3
NO3
FraEuI1c_3196
FraEuI1c_4521
Sulfur Analogs
TehA
TeO3
SeO4
TeO3
Te0
CysPUWA
FraEuI1c_6602
SeO3
SeO3
Se0
FraEuI1c_1092 - 95
SO4
MoO4
TerC
NirBD
TeO3
CrO4
FraEuI1c_3871
SulP
FraEuI1c_4513 - 14
FraEuI1c_4031
SO4
SirA
MoO4
FraEuI1c_0605
FraEuI1c_6285
CrO4
ChrA
CrO4
FraEuI1c_4414
Molybdenum
SeO3
SeO4
SeO4
SO4
ModABC
TauZ
WO4
SorA
FraEuI1c_7020 - 22
MoO4
CrO4
FraEuI1c_4373
MoO4
FraEuI1c_6626
OUT
OUT
Strain Info: Sym+, N2-fix-, Nir, Cor, Cdr, Pbr, Ser, Cur, AsO4s
