# Supplementary material for: The ins and outs of metal homeostasis by the root nodule actinobacterium Frankia
Source: BMC Genomics. 2014 Dec 12;15:1092. doi: 10.1186/1471-2164-15-1092 (PMC4531530; doi:10.1186/1471-2164-15-1092)
Supplement: Supplementary file 17 — Additional file 17: Frankia sp. strain CN3 metal homeostasis mechanisms. Schematic diagram of known and putative metal homeostasis systems in Frankia sp. strain CN3. Loci containing identifying domains (see Additional file 10) for metal ion uptake transporters, chaperones, modification enzymes, efflux transporters, and surface binding protein and efflux systems are shown (left to right) with arrows to indicate the flow of metals through the cell. Information at the bottom indicates whether the strain is symbiotic with host plants (Sym+/-), is a diazotroph (N2-fix+/-), and whether the strain is resistant (r) or sensitive (s) to a particular metal. * = DRAFT. (PPT 201 KB) [file 12864_2014_7073_MOESM17_ESM.ppt]

## Slide 1
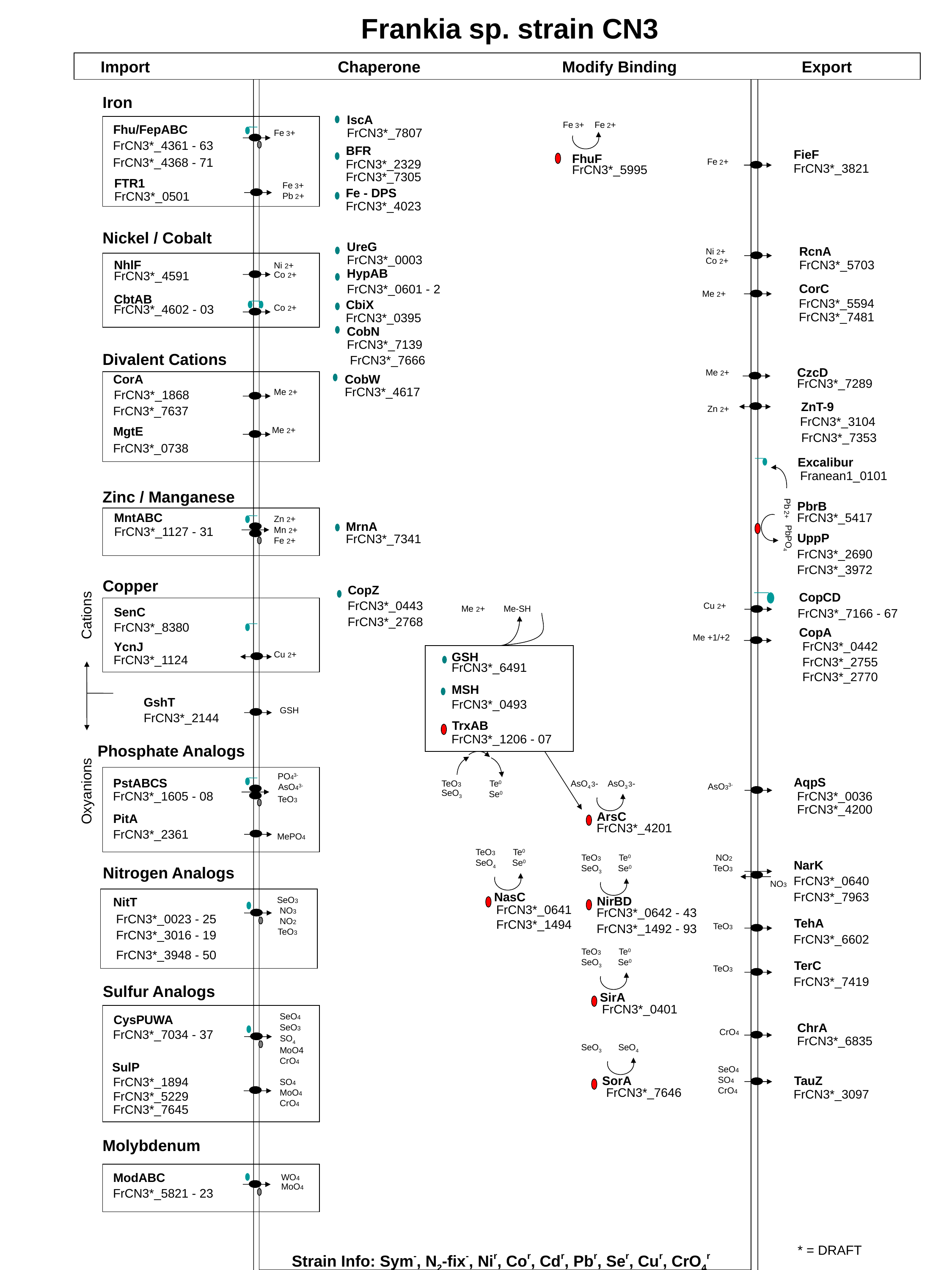

Frankia sp. strain CN3
 Import	 Chaperone 	 Modify Binding 	 Export
Iron
IscA
Fe 3+
Fe 2+
Fhu/FepABC
FrCN3*_7807
Fe 3+
FrCN3*_4361 - 63
BFR
FieF
FhuF
FrCN3*_4368 - 71
Fe 2+
FrCN3*_2329
FrCN3*_5995
FrCN3*_3821
FrCN3*_7305
FTR1
Fe 3+
FrCN3*_0501
Fe - DPS
Pb 2+
FrCN3*_4023
Nickel / Cobalt
UreG
RcnA
Ni 2+
FrCN3*_0003
Co 2+
NhlF
FrCN3*_5703
Ni 2+
HypAB
FrCN3*_4591
Co 2+
FrCN3*_0601 - 2
CorC
Me 2+
CbtAB
FrCN3*_5594
CbiX
FrCN3*_4602 - 03
Co 2+
FrCN3*_7481
FrCN3*_0395
CobN
FrCN3*_7139
Divalent Cations
FrCN3*_7666
CzcD
Me 2+
CobW
CorA
FrCN3*_7289
FrCN3*_4617
Me 2+
FrCN3*_1868
ZnT-9
FrCN3*_7637
Zn 2+
FrCN3*_3104
MgtE
Me 2+
FrCN3*_7353
FrCN3*_0738
Excalibur
Franean1_0101
Zinc / Manganese
Pb 2+
PbPO4
PbrB
MntABC
FrCN3*_5417
Zn 2+
MrnA
FrCN3*_1127 - 31
Mn 2+
UppP
FrCN3*_7341
Fe 2+
FrCN3*_2690
FrCN3*_3972
Copper
CopZ
CopCD
Cations
FrCN3*_0443
Cu 2+
Me 2+
Me-SH
SenC
FrCN3*_7166 - 67
FrCN3*_2768
FrCN3*_8380
CopA
Me +1/+2
FrCN3*_0442
YcnJ
GSH
Cu 2+
FrCN3*_1124
FrCN3*_2755
FrCN3*_6491
FrCN3*_2770
MSH
GshT
FrCN3*_0493
GSH
FrCN3*_2144
TrxAB
FrCN3*_1206 - 07
Phosphate Analogs
TeO3
Te0
SeO3
Se0
PO43-
AqpS
PstABCS
AsO4 3-
AsO3 3-
Oxyanions
AsO33-
AsO43-
FrCN3*_1605 - 08
FrCN3*_0036
TeO3
FrCN3*_4200
ArsC
PitA
FrCN3*_4201
FrCN3*_2361
MePO4
TeO3
Te0
TeO3
Te0
NO2
SeO4
Se0
NarK
Nitrogen Analogs
SeO3
Se0
TeO3
FrCN3*_0640
NO3
NasC
FrCN3*_7963
NirBD
NitT
SeO3
FrCN3*_0641
NO3
FrCN3*_0642 - 43
FrCN3*_0023 - 25
NO2
TehA
FrCN3*_1494
FrCN3*_1492 - 93
TeO3
TeO3
FrCN3*_3016 - 19
FrCN3*_6602
TeO3
Te0
FrCN3*_3948 - 50
SeO3
Se0
TerC
TeO3
FrCN3*_7419
Sulfur Analogs
SirA
FrCN3*_0401
SeO4
CysPUWA
ChrA
SeO3
FrCN3*_7034 - 37
CrO4
SO4
FrCN3*_6835
SeO3
SeO4
MoO4
CrO4
SulP
SeO4
TauZ
SorA
FrCN3*_1894
SO4
SO4
FrCN3*_7646
CrO4
FrCN3*_3097
MoO4
FrCN3*_5229
CrO4
FrCN3*_7645
Molybdenum
ModABC
WO4
MoO4
FrCN3*_5821 - 23
* = DRAFT
Strain Info: Sym-, N2-fix-, Nir, Cor, Cdr, Pbr, Ser, Cur, CrO4r
