# Supplementary material for: The ins and outs of metal homeostasis by the root nodule actinobacterium Frankia
Source: BMC Genomics. 2014 Dec 12;15:1092. doi: 10.1186/1471-2164-15-1092 (PMC4531530; doi:10.1186/1471-2164-15-1092)
Supplement: Supplementary file 18 — Additional file 18: Frankia sp. strain DC12 metal homeostasis mechanisms. Schematic diagram of known and putative metal homeostasis systems in Frankia sp. strain DC12. Loci containing identifying domains (see Additional file 10) for metal ion uptake transporters, chaperones, modification enzymes, efflux transporters, and surface binding protein and efflux systems are shown (left to right) with arrows to indicate the flow of metals through the cell. Information at the bottom indicates whether the strain is symbiotic with host plants (Sym+/-), is a diazotroph (N2-fix+/-), and whether the strain is resistant (r) or sensitive (s) to a particular metal. * = DRAFT. (PPT 182 KB) [file 12864_2014_7073_MOESM18_ESM.ppt]

## Slide 1
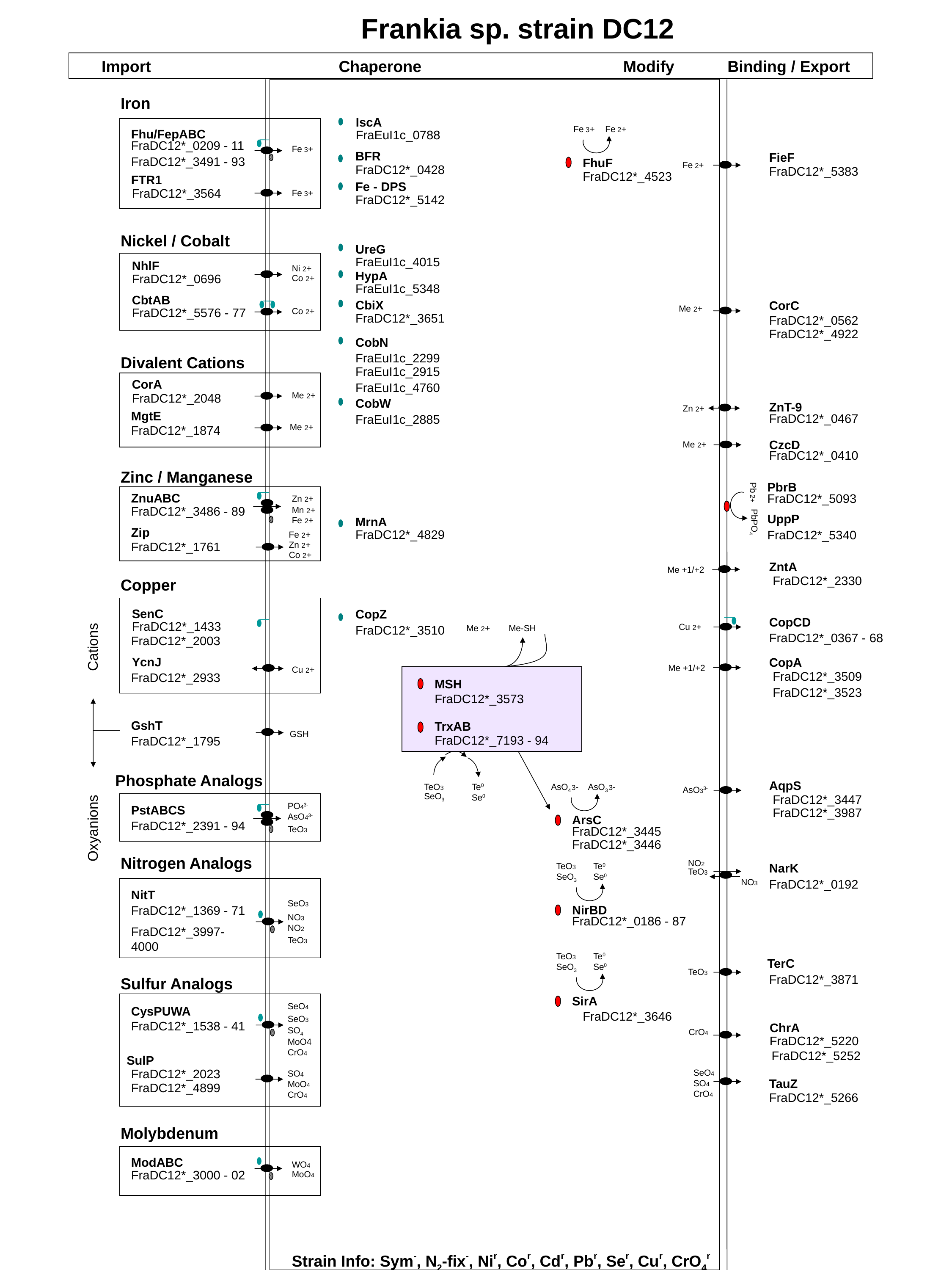

Frankia sp. strain DC12
 Import	 Chaperone 		 Modify 	Binding / Export
Iron
IscA
Fe 3+
Fe 2+
FraEuI1c_0788
Fhu/FepABC
FraDC12*_0209 - 11
Fe 3+
BFR
FieF
FraDC12*_3491 - 93
FhuF
Fe 2+
FraDC12*_0428
FraDC12*_5383
FraDC12*_4523
FTR1
Fe - DPS
FraDC12*_3564
Fe 3+
FraDC12*_5142
Nickel / Cobalt
UreG
FraEuI1c_4015
NhlF
Ni 2+
HypA
FraDC12*_0696
Co 2+
FraEuI1c_5348
CbtAB
CbiX
CorC
Me 2+
FraDC12*_5576 - 77
Co 2+
FraDC12*_3651
FraDC12*_0562
FraDC12*_4922
CobN
FraEuI1c_2299
Divalent Cations
FraEuI1c_2915
CorA
FraEuI1c_4760
Me 2+
FraDC12*_2048
CobW
ZnT-9
Zn 2+
MgtE
FraDC12*_0467
FraEuI1c_2885
Me 2+
FraDC12*_1874
CzcD
Me 2+
FraDC12*_0410
Zinc / Manganese
Pb 2+
PbPO4
PbrB
FraDC12*_5093
ZnuABC
Zn 2+
FraDC12*_3486 - 89
Mn 2+
UppP
MrnA
Fe 2+
FraDC12*_4829
Zip
FraDC12*_5340
Fe 2+
FraDC12*_1761
Zn 2+
Co 2+
ZntA
Me +1/+2
FraDC12*_2330
Copper
CopZ
SenC
CopCD
FraDC12*_1433
Cu 2+
FraDC12*_3510
Me 2+
Me-SH
Cations
FraDC12*_0367 - 68
FraDC12*_2003
YcnJ
CopA
Me +1/+2
Cu 2+
FraDC12*_3509
FraDC12*_2933
MSH
FraDC12*_3523
FraDC12*_3573
GshT
TrxAB
GSH
FraDC12*_7193 - 94
FraDC12*_1795
TeO3
Te0
SeO3
Se0
Phosphate Analogs
AqpS
AsO4 3-
AsO3 3-
AsO33-
FraDC12*_3447
PO43-
FraDC12*_3987
PstABCS
AsO43-
ArsC
FraDC12*_2391 - 94
Oxyanions
TeO3
FraDC12*_3445
FraDC12*_3446
Nitrogen Analogs
NO2
TeO3
Te0
NarK
TeO3
SeO3
Se0
NO3
FraDC12*_0192
NitT
SeO3
NirBD
FraDC12*_1369 - 71
NO3
FraDC12*_0186 - 87
NO2
FraDC12*_3997- 4000
TeO3
TeO3
Te0
TerC
SeO3
Se0
TeO3
FraDC12*_3871
Sulfur Analogs
SirA
SeO4
CysPUWA
FraDC12*_3646
SeO3
FraDC12*_1538 - 41
ChrA
SO4
CrO4
FraDC12*_5220
MoO4
CrO4
FraDC12*_5252
SulP
FraDC12*_2023
SeO4
SO4
TauZ
SO4
MoO4
FraDC12*_4899
CrO4
CrO4
FraDC12*_5266
Molybdenum
ModABC
WO4
FraDC12*_3000 - 02
MoO4
Strain Info: Sym-, N2-fix-, Nir, Cor, Cdr, Pbr, Ser, Cur, CrO4r
