# Supplementary material for: The ins and outs of metal homeostasis by the root nodule actinobacterium Frankia
Source: BMC Genomics. 2014 Dec 12;15:1092. doi: 10.1186/1471-2164-15-1092 (PMC4531530; doi:10.1186/1471-2164-15-1092)
Supplement: Supplementary file 20 — Additional file 20: Phylogenetic analysis of the MerR family proteins in Frankia. Neighbor-joining tree of Clustal Ω aligned MerR proteins (COG0789) containing protein sequences from Frankia, Bacillus subtilis subtilis 168, Escherichia coli K12-W3110, and Cupriavidus metallidurans CH34. The ten clusters of Frankia MerR proteins comprise two distinct group of metal and non-metal regulators. Underlined genes were upregulated with the indicated metals from the compiled gene array studies (see Additional file 1). Asterisk indicates the conservation of a motif. ([LIV][SADG][DER].....[DEGA].{3,4}[^S].[LIV][DR][FHDCY]Y.{3,4}GL[LIVMF].*[^Q][GR].[FY]..[^H]) derived from protein sequence alignment of the upregulated MerR array genes. (PPTX 406 KB) [file 12864_2014_7073_MOESM20_ESM.pptx]

## Slide 1
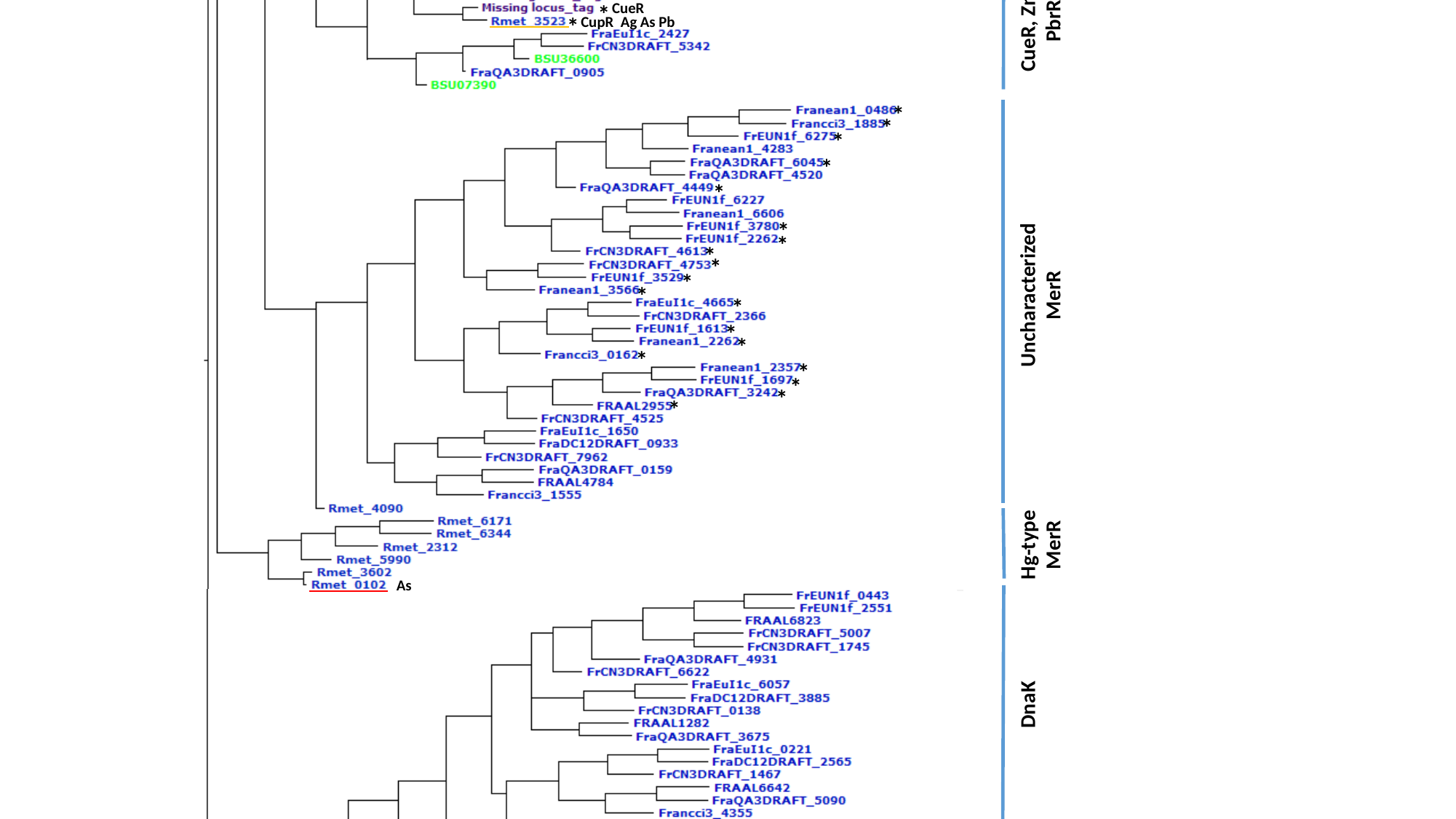

Uncharacterized MerR
CueR, MerD
Uncharacterized MerR
CueR, ZntR, PbrR
Uncharacterized MerR
Hg-type MerR
DnaK
MlrA
YwkC
GlnR
SoxR
*
*
*
*
*
MerD Hg Cd Pb
MerD Hg Cd Pb
*
*
CueR
*
*
*
*
*
*
*
*
*
*
*
*
Metal Regulators
*
*
*
PbR Pb Co As
ZntR
CueR
*
*
CupR Ag As Pb
*
*
*
*
*
*
*
*
*
*
*
*
*
*
*
*
*
*
*
As
Non-Metal Regulators
