# Supplementary material for: The ins and outs of metal homeostasis by the root nodule actinobacterium Frankia
Source: BMC Genomics. 2014 Dec 12;15:1092. doi: 10.1186/1471-2164-15-1092 (PMC4531530; doi:10.1186/1471-2164-15-1092)
Supplement: Supplementary file 21 — Additional file 21: Phylogenetic analysis of the ArsR family proteins in Frankia. Neighbor-joining tree of Clustal Ω aligned ArsR proteins (COG0640) containing protein sequences from Frankia, upregulated genes from the compiled array data (Additional file 1), and characterized ArsR-family proteins. The metals that induced up-regulation of the gene from the array studies are listed next to the gene. The five clusters of Frankia ArsR proteins include 1. a group with no similarity to characterized ArsR proteins, 2. a group that is in a potential operon with the cation diffusion facilitor (CzcD), 3. a group that is in a potential operon with the activator of heat shock 90 ATPase protein (AHSA), 4. a group highly similar to the M. tuberculosis nickel responsive repressor (NmtR), and 5. a group in a potential ars operon with other arsenic resistance genes. (PPTX 387 KB) [file 12864_2014_7073_MOESM21_ESM.pptx]

## Slide 1
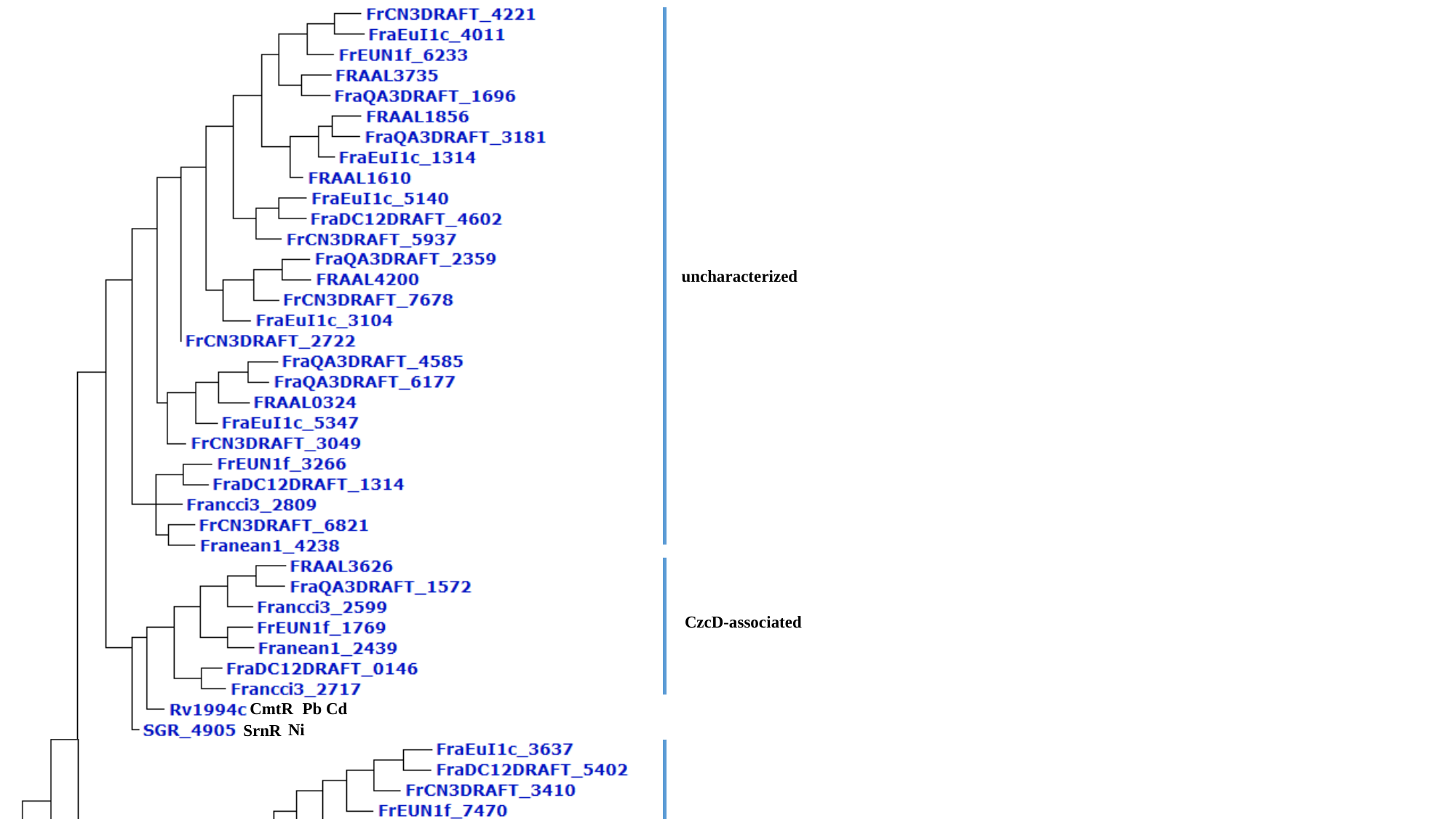

uncharacterized
CzcD-associated
CmtR
Pb Cd
Ni
SrnR
Cu
AHSA-associated
Cu
Cd As Zn Pb Se
Cd
Cd
Fe Co Mn
Cd
Cd
NmtR-like
NmtR
Cu
Fe Mn Co
Se
Ars-associated
ArsR
ArsR
Fe Mn Co
Cu
Cr
CadC
CadC
As Cd Ag Zn Cu Ni
CzrA
BxmR
ZiaR
SmtB
