# Supplementary material for: The ins and outs of metal homeostasis by the root nodule actinobacterium Frankia
Source: BMC Genomics. 2014 Dec 12;15:1092. doi: 10.1186/1471-2164-15-1092 (PMC4531530; doi:10.1186/1471-2164-15-1092)
Supplement: Supplementary file 23 — Additional file 23: Phylogenetic analysis of the Fur family proteins in Frankia. Neighbor-joining tree of Clustal Ω aligned MerR proteins (KO:K03711) containing protein sequences from Frankia, and the 18 comparative organisms (Additional file 10). Representative neighborhoods are included to demonstrate synteny of the 4 regulators (Fur, Nur, PerR, Zur) in Frankia. Arrows indicate the regulators and their targets. Asterisks indicate characterized proteins. (PPTX 874 KB) [file 12864_2014_7073_MOESM23_ESM.pptx]

## Slide 1
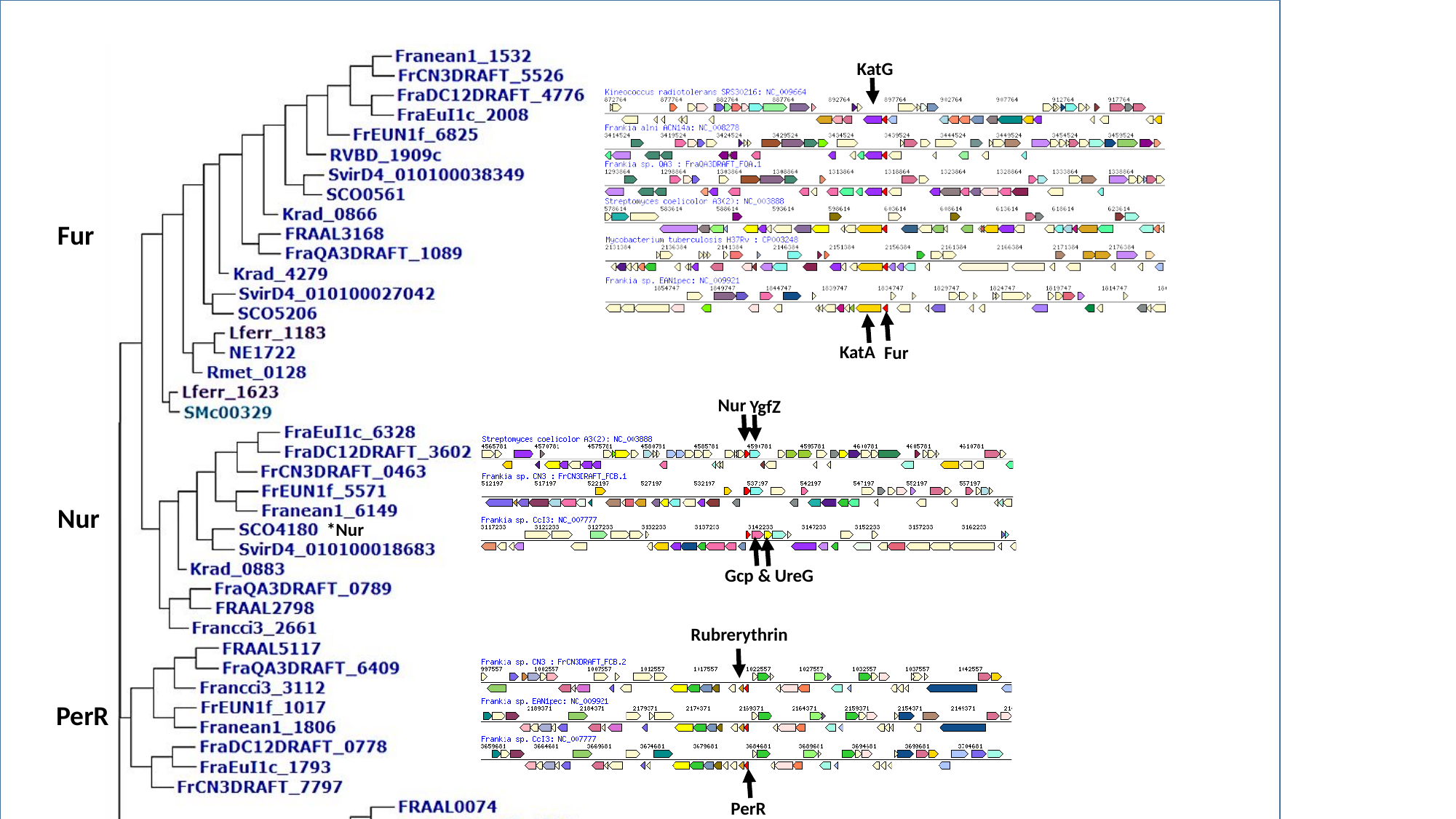

KatG
Fur
KatA
Fur
Nur
YgfZ
Nur
*Nur
Gcp & UreG
Rubrerythrin
PerR
PerR
ArsR
Zur
Zur
ZnuABC
*Zur
*Zur
*Zur
*Fur
*Mur
