# Supplementary material for: The ins and outs of metal homeostasis by the root nodule actinobacterium Frankia
Source: BMC Genomics. 2014 Dec 12;15:1092. doi: 10.1186/1471-2164-15-1092 (PMC4531530; doi:10.1186/1471-2164-15-1092)
Supplement: Supplementary file 24 — Additional file 24: Methodology workflow chart. Breakdown of the process for identification of metal homeostasis mechanisms used in this study. (PPT 61 KB) [file 12864_2014_7073_MOESM24_ESM.ppt]

## Slide 1
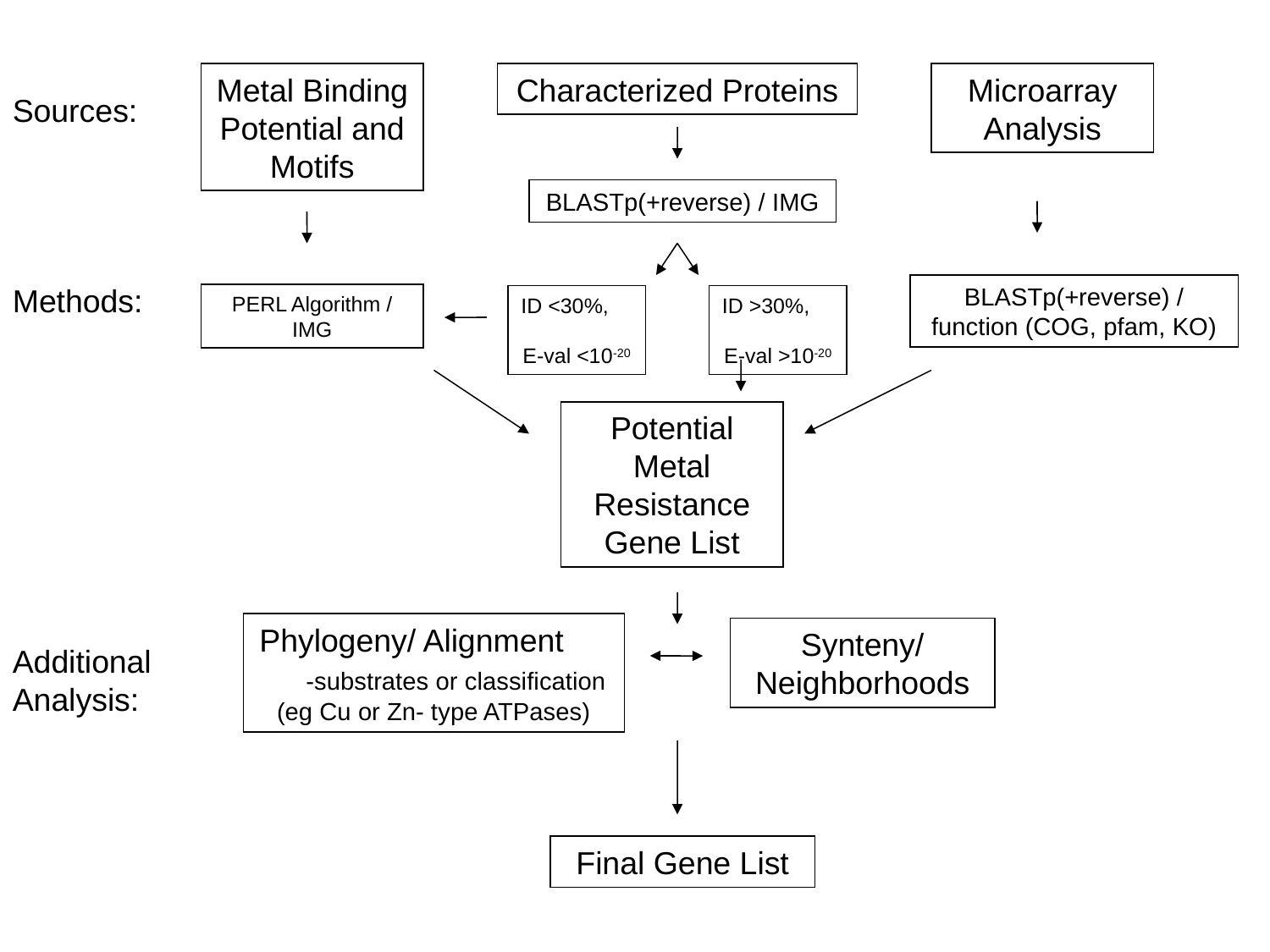

Metal Binding Potential and Motifs
Characterized Proteins
Microarray Analysis
Sources:
BLASTp(+reverse) / IMG
Methods:
BLASTp(+reverse) / function (COG, pfam, KO)
PERL Algorithm / IMG
ID <30%, E-val <10-20
ID >30%, E-val >10-20
Potential Metal Resistance Gene List
Phylogeny/ Alignment -substrates or classification (eg Cu or Zn- type ATPases)
Synteny/ Neighborhoods
Additional Analysis:
Final Gene List
